# RPL41 stabilizes ribosome dynamics and supports long-protein homeostasis in mammals

**DOI:** 10.1093/nar/gkag797

**Published:** 2026-08-19

**Authors:** Mina Hirata, Maho Fujino, Kazuya Ichihara, Ronghao Tang, Momoko Narita, Wakana Iwasaki, Chisa Shiraishi, Taichi Shiraishi, Atsushi Hatano, Toru Suzuki, Tokichiro Abe, Tomoyo Takami, Emi Mishiro-Sato, Atsushi Toyoda, Masaki Matsumoto, Yoshikazu Tanaka, Takuhiro Ito, Toshifumi Inada, Keiichi I Nakayama, Takeshi Yokoyama, Akinobu Matsumoto

**Affiliations:** Department of Biological Science, Division of Biological Science, Graduate School of Science, Nagoya University, Nagoya 464-8602, Japan; Department of Molecular and Chemical Life Sciences, Graduate School of Life Sciences, Tohoku University, Sendai, Miyagi 980-8577, Japan; Department of Biological Science, Division of Biological Science, Graduate School of Science, Nagoya University, Nagoya 464-8602, Japan; Division of Cell Biology, Medical Institute of Bioregulation, Kyushu University, Fukuoka 812-8582, Japan; Department of Basic Medical Sciences, School of Medicine, Tsinghua University, Beijing 100084, China; Division of RNA and Gene Regulation, Institute of Medical Science, The University of Tokyo, Minato-Ku 108-8639, Japan; Laboratory for Translation Structural Biology, RIKEN Center for Integrative Medical Sciences, Yokohama 230-0045, Japan; Department of Biological Science, Division of Biological Science, Graduate School of Science, Nagoya University, Nagoya 464-8602, Japan; Department of Biological Science, Division of Biological Science, Graduate School of Science, Nagoya University, Nagoya 464-8602, Japan; Department of Omics and Systems Biology, Graduate School of Medical and Dental Sciences, Niigata University, Niigata 951-8510, Japan; Division of RNA and Gene Regulation, Institute of Medical Science, The University of Tokyo, Minato-Ku 108-8639, Japan; Department of Omics and Systems Biology, Graduate School of Medical and Dental Sciences, Niigata University, Niigata 951-8510, Japan; Department of Omics and Systems Biology, Graduate School of Medical and Dental Sciences, Niigata University, Niigata 951-8510, Japan; Institute of Transformative Bio-Molecules (WPI-ITbM), Nagoya University, Nagoya 464-8601, Japan; Advanced Genomics Center, National Institute of Genetics, Shizuoka 411-8540, Japan; Department of Omics and Systems Biology, Graduate School of Medical and Dental Sciences, Niigata University, Niigata 951-8510, Japan; Department of Molecular and Chemical Life Sciences, Graduate School of Life Sciences, Tohoku University, Sendai, Miyagi 980-8577, Japan; Laboratory for Translation Structural Biology, RIKEN Center for Integrative Medical Sciences, Yokohama 230-0045, Japan; Division of RNA and Gene Regulation, Institute of Medical Science, The University of Tokyo, Minato-Ku 108-8639, Japan; Division of Cell Biology, Medical Institute of Bioregulation, Kyushu University, Fukuoka 812-8582, Japan; Anticancer Strategies Laboratory, Advanced Research Initiative, Institute of Science Tokyo, Tokyo 113-8510, Japan; Department of Molecular and Chemical Life Sciences, Graduate School of Life Sciences, Tohoku University, Sendai, Miyagi 980-8577, Japan; Department of Aquaculture Life Science, Graduate School of Fisheries Sciences, Hokkaido University, Hakodate, Hokkaido 041-8611, Japan; Department of Biological Science, Division of Biological Science, Graduate School of Science, Nagoya University, Nagoya 464-8602, Japan; Center for Human Disease Genomics and Medical Science, Research Institute of Environmental Medicine, Nagoya University, Nagoya 464-8601, Japan

## Abstract

Ribosomal protein L41 (RPL41 or eL41) is the smallest ribosomal protein and forms the eukaryote-specific bridge, eB14, near the decoding center; however, its role in mammalian translation remains unclear. In this study, we established RPL41-deficient models of human HEK293T cells and mice to define its function. Cryo-electron microscopy revealed that RPL41 constrains intersubunit conformational dynamics without inducing major local static rearrangements. Loss of RPL41 altered A-site dynamics, slowed elongation, modestly increased amino acid misincorporation, and modestly enhanced readthrough of collision-inducing reporter sequences. Quantitative proteomic analysis suggested that these translational defects compromise long-protein homeostasis, as evidenced by increased insolubility and reduced abundance of long proteins. *In vivo*, *Rpl41^−/−^* mice were viable but exhibited growth retardation and decreased abundance of long proteins in tissues. Our findings reveal a conserved role for RPL41 in maintaining ribosome dynamics and translational fidelity, indicating that RPL41 supports ribosome function and long-protein homeostasis in mammals.

## Graphical Abstract

**Graphical Abstract:**
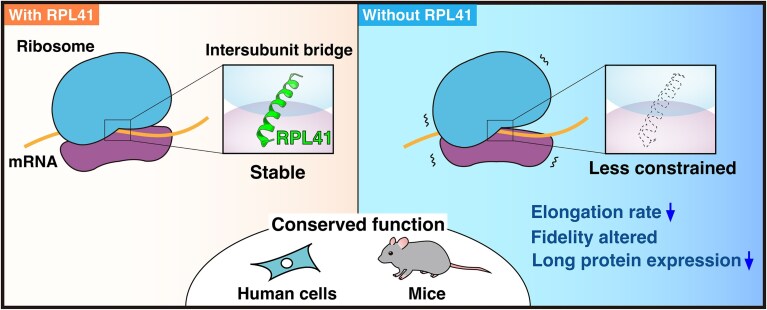

## Introduction

In eukaryotes, ribosomes consist of small 40S subunit and large 60S subunits. Together, these subunits form an 80S ribosome comprising ~80 ribosomal proteins (RPs) and four ribosomal RNAs (rRNAs) [1]. Traditionally, ribosomes were considered to have a uniform composition. However, accumulating evidence has led to the emergence of the concept of ribosome heterogeneity, which posits that variations in ribosomal composition or post-translational modifications can result in the selective translation of specific subsets of messenger RNAs (mRNAs) [2–7]. Although most core RPs are consistently incorporated into ribosomes, functional ribosomes lacking specific RPs have been identified. For example, ribosomal subpopulations deficient in RPS25/eS25 or RPL10A/uL1 preferentially translate distinct mRNA subsets [2]. Furthermore, ribosomes associated with the mitochondrial outer membrane are enriched in complexes deficient in RPS25 and RPL29/eL29, which show preferential association with mRNAs involved in metabolic processes [8].

Homozygous knockout (KO) mouse models targeting individual ribosomal subunits have provided important insights into the molecular mechanisms underlying ribosomal heterogeneity and their physiological and pathological roles. Although loss of most core RP (e.g. Rpl3/uL3, Rpl10a, Rps19/eL19, and Rps12/eS12) results in embryonic lethality [9–12], Rpl29 KO mice are viable [13–16]. These mice exhibit intrauterine growth retardation, reduced placental size, delayed ossification, and significant postnatal growth delay, with some pups weighing up to 50% less than their wild-type (WT) littermates at weaning. Low birth weight is associated with increased postnatal mortality, contributing to deviations from the expected Mendelian inheritance ratios. Rpl22/eL22 KO mice are also viable, likely because of functional compensation by its paralog, Rpl22l1/eL22L1 [17–20].

RPL41 (also known as eL41) is an RP found in ribosomes of all eukaryotes and some archaea [21]. It is the smallest known RP, comprising only 25 amino acids, of which 17 are basic residues, either arginine or lysine. RPL41 adopts a single α-helix structure. Although initially thought to be a component of the 60S, it is now recognized as an integral part of the 40S. RPL41 contributes to the formation of the eukaryote-specific bridge eB14, which connects the two subunits near the decoding center [22, 23]. Importantly, RPL41 is one of the few non-essential RPs [24, 25]. Yeast cells lacking RPL41 remain viable and exhibit growth comparable to that of WT cells, in contrast to the lethality observed in yeasts lacking most other RPs. Although the absence of RPL41 does not markedly affect polysome formation, it is associated with reduced peptidyl transferase activity [25, 26]. This reduced activity correlates with an increased rate of programmed −1 ribosomal frameshifting, revealing the important role of RPL41 in maintaining translational fidelity in yeast [27].

However, the role of RPL41 in mammalian cells remains unclear. To address this, we established RPL41-deficient models in human HEK293T cells and mice. In HEK293T cells, the loss of RPL41 resulted in diverse translational abnormalities, including altered polysome profiles, reduced elongation rates, a modest increase in readthrough of collision-inducing reporter sequences, and increased amino acid misincorporation, collectively affecting the proteome. *In vivo*, RPL41 KO mice were viable but exhibited growth retardation and reduced expression of long proteins in tissues. These findings revealed the molecular role of the non-essential RP RPL41 in regulating ribosome dynamics and underscored its physiological importance in mammals. More broadly, as eukaryotic proteomes are enriched in long and multi-domain proteins compared with prokaryotes, the conservation of RPL41 in eukaryotes may reflect an adaptation to these translational demands.

## Materials and methods

### Cell lines and culture conditions

HEK293T (RCB2202) cells were obtained from the RIKEN BioResource Center through the National Bio-Resource Project of MEXT, Japan. Cells were maintained in Dulbecco’s modified Eagle’s medium (DMEM) supplemented with 10% fetal bovine serum (FBS) and penicillin–streptomycin (100 U/ml) at 37°C in 5% CO_2_. 293FT cells (R700-07; Thermo Fisher Scientific) were cultured in DMEM supplemented with 10% FBS, 1× Non-Essential Amino Acids (Thermo Fisher Scientific), and penicillin–streptomycin (100 U/ml). The cell lines were tested for mycoplasma contamination using the MycoAlert Mycoplasma Detection Kit (Lonza).

### Animals

All animal experiments were approved by the Animal Ethics Committees of Kyushu University (A20-169-0, A21-271-0, and A22-013-0) and Nagoya University (S230016, S240004, and S250002) and were conducted in accordance with institutional guidelines and regulations for animal care and use. To generate *Rpl41*^–/–^ mice, ribonucleoproteins were prepared by mixing the clustered regularly interspaced short palindromic repeats (CRISPR) RNA and transactivating CRISPR RNA with recombinant CRISPR-associated protein 9 (Cas9) (Integrated DNA Technologies) (Supplementary Table S1). Mouse zygotes of the C57BL/6J strain were subjected to electroporation with each ribonucleoprotein complex. All mice were housed in the specific pathogen-free animal facility at Kyushu University and Nagoya University in accordance with institutional guidelines under the following conditions: ambient temperature of 22°C, 50%–60% humidity, 12-h-dark/12-h-light cycle, and free access to water and rodent chow CA-1 (CLEA Japan).

### Generation of RPL41- or ASCC2-deficient cell lines

Sense and antisense oligonucleotides (Supplementary Table S1) encoding single-guide RNAs targeting human RPL41 or ASCC2 were cloned into the pSpCas9(BB)-2A-Puro (PX459) V2.0 vector (Addgene plasmid #62988) [28]. HEK293T cells were seeded in 6-cm dishes at ∼50% confluence and transfected with single-guided RNA-encoding PX459 plasmids using TransIT-LT1 (Mirus Bio). Twenty-four hours post-transfection, cells were trypsinized and reseeded at a 1:5 ratio into medium containing 1 µg/ml puromycin to select transfected cells. After 48 h of puromycin selection, the surviving single cells were plated in 96-well plates and expanded for 10–14 days. Monoclonal populations were screened using immunoblotting to identify RPL41 or ASCC2 KO clones, and successful KOs were further validated by genomic sequencing.

### Immunoblot analysis

Protein samples were subjected to sodium dodecyl sulfate (SDS)–polyacrylamide gel electrophoresis on SuperSep Ace 5%–20% (Fujifilm). Membranes were incubated consecutively with primary antibodies [RPL41 (Thermo Fisher Scientific, PA5-68075) and HSP90 (BD Biosciences, 610419)] and horseradish peroxidase-conjugated secondary antibodies (Promega), and signals were visualized using SuperSignal West (Thermo Fisher Scientific) reagents on a ChemiDoc imaging system (Bio-Rad).

### OP-puro incorporation assay

HEK293T cells were incubated with 50 μM OP-puro for the indicated times at 37°C, transferred immediately onto ice, and washed twice with phosphate-buffered saline (PBS) containing 3% bovine serum albumin. The cells were fixed with 1% formaldehyde on ice for 15 min, permeabilized with PBS containing 0.1% saponin and 3% FBS for 5 min at room temperature, and subjected to Click-iT labeling with Alexa Fluor 488 azide (3 μM, Invitrogen) for 30 min at room temperature in the dark. After washing, cells were resuspended in PBS and analyzed by FACSLyric (BD Biosciences). Mean fluorescence intensity was used to quantify OP-puro incorporation.

### Cell proliferation assay

HEK293T cells were seeded onto poly-L-lysine-coated plates and harvested on days 1–5 after seeding for analysis. At each time point, cells were washed with PBS and fixed with 4.1% glutaraldehyde in PBS (Fujifilm) at room temperature for 30 min. After washing with PBS and air drying, the cells were stained with 1% crystal violet (Merck) at room temperature for 30 min. Excess staining was removed by washing with PBS, followed by destaining with 10% acetic acid in PBS for 10 min at room temperature. The plates were then washed with PBS and air-dried, and the bound dye was solubilized with 33% acetic acid. The resulting solution was transferred to a 96-well clear microplate, and absorbance was measured at 600 nm using a GloMax Explorer Multimode Microplate Reader (Promega).

### Sucrose density gradient analysis

Cells were lysed in lysis buffer [50 mM Tris–HCl (pH 7.5), 150 mM NaCl, 5 mM MgCl_2_, 1 mM dithiothreitol (DTT), 1% Triton X-100, and ethylenediaminetetraacetic acid-free protease inhibitor] supplemented with cycloheximide (CHX) (100 μg/ml) and SUPERase•In RNase inhibitor (10 U/ml, Invitrogen), and the lysates were incubated for 10 min on ice and then centrifuged at 20 380 × *g* for 10 min at 4°C. The resulting supernatants were loaded on a 15%–50% sucrose gradient buffer [20 mM Tris–HCl (pH 7.5), 150 mM NaCl, 5 mM MgCl_2_, 1 mM DTT, CHX (100 μg/ml), and SUPERase•In (10 U/ml)] and centrifuged at 36 000 rpm (163 000 × *g*) for 2 h or 35 000 rpm (154 000 × *g*) for 5 h at 4°C in a Hitachi P40ST rotor. The gradient was fractionated using a TRIAX gradient profiling system (BioComp) and an AC-5700P microcollector (ATTO). Polysome profiles were determined by measuring the absorbance at 260 nm.

### Lentivirus production and infection

CSII-CMV-MCS-IRES2-Bsd (RDB04385), pCAG-HIVgp (RDB04394), and pCMV-VSV-G-RSV-Rev (RDB04393) were kindly provided by Dr Hiroyuki Miyoshi (RIKEN BioResource Center; Ibaraki, Japan) [29]. pmGFP-P2A-K0-P2A-RFP and pmGFP-P2A-K(AAA)20-P2A-RFP were gifts from Dr Ramanujan Hegde (Addgene plasmids #105686 and #105688) [30]. A human XBP1u complementary DNA (cDNA) fragment (amino acids 209–261) was amplified using polymerase chain reaction (PCR) and inserted into pmGFP-P2A-K0-P2A-RFP at the KpnI–SalI sites, replacing the K0 sequence, to generate pmGFP-P2A-XBP1u-P2A-RFP. Lentiviral vectors were constructed by subcloning the GFP-P2A-K0/K20/XBP1u-P2A-RFP cassettes into multiple cloning sites of CSII-CMV-MCS-IRES2-Bsd. The resulting constructs, together with pCAG-HIVgp and pCMV-VSV-G-RSV-Rev, were co-transfected into 293FT cells using Lipofectamine 2000 (Thermo Fisher Scientific). After transfection, the medium was replaced with pre-warmed DMEM supplemented with 10% FBS. Forty-eight hours later, lentivirus-containing culture supernatants were collected and filtered through 0.45 μm filters (Millipore, MILLEX GV). Viral solutions were supplemented with 5 μg/ml polybrene (Sigma) and applied to HEK293T cells. Forty-eight hours post-infection, cells were trypsinized, and one-fifth of the infected population was reseeded onto new 10 cm plates and cultured in medium containing 10 μg/ml blasticidin-S (Wako) for 48 h to select infected cells.

### Flow cytometry

HEK293T cells stably transduced with GFP-P2A-K0/K20/XBP1u-P2A-RFP cassettes were harvested via trypsinization, washed twice with PBS, and analyzed using a CytoFLEX S flow cytometer (Beckman Coulter). Data were processed using FlowJo software v10.10 (FlowJo). Cells within the forward scatter range of 300 000–1 000 000 and the side scatter range of 250 000–2 000 000 were used for the analysis. The threshold for the green fluorescent protein (GFP) signals was set between 1 × 10^5^ and 1 × 10^8^.

### Luciferase reporter assay

Reporter plasmids were generated by cloning the DNA sequences of Renilla luciferase (Rluc) and Firefly luciferase (Fluc) into the pcDNA3 vector (Invitrogen) and linked by either a control sequence, a frameshift-inducing sequence, or a sequence containing a stop codon (see Supplementary Table S1 for linker sequences) [31]. For the misincorporation assay, a reporter construct was generated using the control linker sequence to fuse Rluc and Fluc, with a point mutation introduced into Fluc that substituted histidine 245 with arginine. Plasmids were transfected into cells using the X-tremeGENE 9 DNA Transfection Reagent (Merck). For experiments with bengamide B (Santa Cruz Biotechnology), the compound was added to the culture medium 2 h after transfection at a final concentration of 0.1 μM. Cells were harvested after 24 h of incubation, and luciferase activity was measured using the Dual-Luciferase Reporter Assay System (Promega) on a GloMax Explorer Multimode Microplate Reader (Promega).

### Histopathologic analysis

Tissues were fixed with 4% paraformaldehyde in PBS, embedded in paraffin, sectioned at 4 µm using a microtome, and stained with hematoxylin and eosin. Sections were examined under a BZ-X700 microscope (Keyence).

### Ribosome purification, grid preparation, and cryo-EM data collection

Human 80S ribosomes were purified from WT and RPL41-deficient HEK293T cells under the same conditions. Cells from 10 culture dishes at ∼80% confluence were harvested by scraping and resuspended in buffer containing 20 mM HEPES (pH 7.4), 100 mM KOAc, and 7.5 mM Mg(OAc)_2_. Suspensions were homogenized using a syringe and clarified via centrifugation at 20 000 × *g* for 10 min at 4°C. Supernatants were layered onto 10%–40% sucrose gradients prepared in the same buffer and centrifuged at 28 000 rpm for 4.5 h at 4°C in an SW28 rotor (Beckman Coulter). The gradients were fractionated from top to bottom using a Gradient Station (BioComp). For cryo-electron microscopy (cryo-EM), 3 µl of aliquots of purified 80S ribosomes (50 nM) were applied to glow-discharged Quantifoil R1.2/1.3 200-mesh Cu grids previously coated with a continuous amorphous carbon film using a JEE-420 vacuum evaporator (JEOL). The grids were blotted with filter paper and plunge-frozen in liquid ethane using a Vitrobot Mark IV (Thermo Fisher Scientific). Data were collected on a CRYO ARM 300 II microscope (JEOL) operated at 300 kV, with images recorded on a K3 direct electron detector (Gatan) at a nominal magnification of 60 000× . The defocus range was −0.5 to −2.2 µm. The total exposure was 40 e/Å^2^ per micrograph, which was divided into 40 movie frames. Automated acquisition was performed using SerialEM [32].

### Single-particle image processing and model building

Patch Motion Correction, Patch Contrast Transfer Function (CTF) Estimation, template-based particle picking, and particle extraction were performed in cryoSPARC v4.4, and the subsequent steps [from two-dimensional (2D) classification onward] in cryoSPARC v4.6 [33]. In total, 7345 (WT; Supplementary Fig. S1A) and 6921 (RPL41-deficient; Supplementary Fig. S2A) movies were motion-corrected, revealing abundant ribosomal particles (WT, Supplementary Fig. S1B; KO, Supplementary Fig. S2B). After CTF estimation, particle picking yielded 277 874 particles (WT) and 373 715 particles (RPL41-deficient) for 2D classification. After 2D classification, clear 80S classes were obtained for both datasets (WT, Supplementary Fig. S1C; KO, Supplementary Fig. S2C). Particles retained for three-dimensional (3D) analysis (255 599 WT and 137 243 KO) were refined to a consensus map using Homogeneous Refinement and subjected to 3D classification using consensus alignments. For the WT dataset, non-rotated (128 805 particles) and rotated (64 802 particles) 80S classes were refined using Homogeneous Refinement, Non-uniform Refinement, Global CTF Refinement, and Local CTF Refinement, yielding reconstructions at 2.48 and 2.69 Å, respectively (Supplementary Fig. S1A and D) [34]. For the RPL41-deficient dataset, 3D classification was performed. Particles were classified into non-rotated 80S (31 234 particles), rotated 80S (30 367 particles), partially rotated 80S (21 228 particles), non-rotated 80S with body roll (18 043 particles), 80S with weak 40S (17 907 particles), and non-rotated 80S with head swivel (15 852 particles). Particles were refined using Homogeneous Refinement, Non-uniform Refinement. Non-rotated 80S and rotated 80S were further refined, yielding maps at 2.83 and 2.91 Å, respectively (Supplementary Fig. S2D). Local resolution was estimated (WT, Supplementary Fig. S1E; KO, Supplementary Fig. S2E). Atomic models were built in UCSF Chimera and Coot using the human 80S structure (PDB 8QOI) as the starting model. For the RPL41-deficient maps, the RPL41 chain was omitted [35–37]. The final models were refined using Phenix.real_space_refine (PHENIX) [38]. Validation statistics are shown in Supplementary Table S2. The figures were prepared using UCSF Chimera and UCSF Chimera X [35, 39].

### Ribo-seq, disome-seq, and harringtonine run-off assay

For the harringtonine run-off assay, cells were treated with 2 μg/ml harringtonine (MedChemExpress) at 37°C for 60, 120, or 180 s, followed by treatment with 100 μg/ml CHX for 1 min to halt translation. The cells were harvested immediately. Cell and tissue lysate supernatants were assayed for RNA concentration using a Qubit RNA BR Assay Kit (Thermo Fisher Scientific), incubated with RNase I (20 U per 10 μg of RNA, Epicentre) for 45 min at 25°C, and placed on ice before the addition of SUPERase•In RNase inhibitor (Invitrogen) at a concentration of 20 U/ml. The samples were then loaded on a 1 M sucrose cushion [containing 20 mM Tris–HCl (pH 7.5), 150 mM NaCl, 5 mM MgCl_2_, SUPERase•In (10 U/ml), 1 mM DTT, and CHX (100 μg/ml)], and the gradients were centrifuged at 100 000 rpm (417 200 × *g*) for 1 h at 4°C in a Beckman TLA110 rotor. Ribosomal footprints were purified using ISOGEN (Fujifilm). The recovered RNA was size-selected using electrophoresis on a 15% polyacrylamide TBE–urea gel (SuperSep RNA, Fujifilm), isolating fragments of 17–34 nt for Ribo-seq and 50–80 nt for disome-seq. The footprint fragments were treated with T4 polynucleotide kinase (New England BioLabs) to repair 2′–3′ cyclic phosphates, and a DNA linker including barcode sequences (NI-810 to NI-817) (Supplementary Table S1) was ligated with the use of T4 RNA ligase 2, truncated K227Q (New England BioLabs). The resulting products were purified on 15% polyacrylamide and TBE–urea gels, and rRNAs were further depleted using a riboPOOL Ribo-seq 96 reaction kit (human/mouse/rat) (Biozym). Reverse transcription was performed using the NI-802 primer (Supplementary Table S1), and the resulting products were purified on a 15% polyacrylamide and TBE–urea gel. The purified cDNAs were circularized with circLigase II (Lucigen), and index sequences were added by PCR amplification with the common primer NI-798 and primers including the index sequences (NI-799 and NI-822 to NI-826) (Supplementary Table S1). Products of the desired size were purified on a 15% polyacrylamide non-denaturing gel (SuperSep DNA, Fujifilm), and libraries were sequenced using NovaSeq (Illumina).

### RNA-seq

Total RNA was extracted from cell lysates using a PureLink RNA Mini Kit (Thermo Fisher Scientific), and the quality of the purified RNA was assessed using a 2100 Bioanalyzer (Agilent). For RNA sequencing (RNA-seq), after mRNA selection via the NEBNext Poly(A) mRNA Magnetic Isolation Module (New England Biolabs), libraries were prepared using the NEBNext Ultra Directional RNA Library Prep Kit for Illumina (New England Biolabs), and the libraries were sequenced with a NovaSeq (Illumina).

### SLAM-seq

To label newly synthesized RNA, cells were incubated for 24 h in culture medium containing 50 μM 4-thiouridine (Merck). The medium was then replaced with fresh medium containing 50 μM uridine, and cells were harvested at 0, 3, 6, and 12 h after the switch. Total RNA was extracted using ISOGEN (NIPPON GENE), according to the manufacturer’s instructions. The purified RNA was alkylated by adding iodoacetamide to a final concentration of 10 mM, followed by incubation at 50°C for 15 min. The reaction was quenched by the addition of DTT to a final concentration of 20 mM. RNA was then purified, and libraries were prepared using the QuantSeq 3′ mRNA-Seq V2 Library Prep Kit (Forward) with Unique Dual Indices (12 nt) (Lexogen). Sequencing was performed using the NovaSeq platform (Illumina).

### Read processing and analysis

Adaptor sequences were trimmed from raw reads using cutadapt. Low-quality reads were discarded using the fastq_quality_trimmer and fastq_quality_filter tools in the FASTX Toolkit. rRNA reads were removed by alignment with human rRNA sequences using STAR, and the remaining reads were aligned to the human or mouse transcriptome (GRCh38.p13 or GRCm38.p6) and genome (hg38 or mm10) using this software. Multimapping reads were allowed. The metagene analysis was performed using custom Python scripts in Numpy (v1.17.3) and Pysam (v0.15.3). The A-site was defined as the position located 15 nt from the start of the 21- and 28-nt reads that showed a 3-nt periodicity. Three-nucleotide periodicity was analyzed using RibORF, and footprints with a score of ≥0.5 were applied to the analysis. Ribo-seq and RNA-seq reads mapped to human coding transcripts were counted using featureCounts (the Subread package). The first and last five codons were excluded from the analysis to avoid atypical footprint counts around the start and stop codons. Ribosome occupancy was calculated as the average of the normalized read counts (RPM) corresponding to the positions of the A sites and was further corrected by the average of the total codon RPMs to obtain the relative ribosome occupancy. The codon-specific dwell times were calculated using RUST [40]. For the harringtonine run-off assay, the codon distance was calculated for each time point. The codon distance was defined as the midpoint between the position of the minimum value and the position where a plateau was reached in the metagene plots spanning 3000 nt from the start codon. The SLAM-seq data were analyzed using the SLAMDUNK analysis pipeline (v0.4.3) [41]. The half-lives of the transcripts were estimated by fitting the CPM values at each time point to an exponential decay function.

### DIA-MS analysis

Cells were lysed in a lysis buffer containing 20 mM Tris–HCl (pH 7.5), 150 mM KCl, 5 mM MgCl_2_, 1% Triton X-100, 1 mM DTT, and a protease inhibitor cocktail (cOmplete, Roche). The lysates were first centrifuged at 500 × *g* for 3 min at 4°C to remove cell debris, and the supernatants were collected. To isolate the insoluble fraction, the supernatants were further centrifuged at 20 000 × *g* for 15 min at 4°C. The resulting pellet was washed three times with PBS, resuspended in SDT buffer containing 4% SDS, 0.1 M DTT, and 100 mM Tris–HCl (pH 7.5), and used for subsequent analysis. For the analysis of the soluble fraction, the supernatants were adjusted to a final protein concentration of 1 mg/ml. Cysteine residues were reduced with 2.5 mM tris(2-carboxyethyl)phosphine hydrochloride (Thermo Fisher Scientific) for 30 min at 37°C and alkylated with 50 mM 2-iodoacetamide (Merck) for 30 min at room temperature. For each sample, 10 μg of protein was subjected to purification and digestion using the SP3 method [42]. The washed beads were then resuspended in 100 μl of 50 mM Tris–HCl (pH 8.0) with 0.2 μg of Lys-C (Fujifilm-Wako) at 37°C for 4 h, followed by digestion with 0.2 μg of trypsin (Promega) and mixed gently at 37°C overnight. The digested sample was acidified with 5 μl of 20% trifluoroacetic acid (TFA). Digested peptides were desalted using GL-Tip SDB (GL Sciences, Tokyo, Japan), evaporated in a SpeedVac concentrator, and redissolved in 0.1% TFA and 2% acetonitrile. LC-MS/MS analysis of the resultant peptides was performed on a nanoLC connected to a Q-Exactive Orbitrap mass spectrometer (Thermo Fisher Scientific) through a nano electrospray ion source (AMR Inc.). The peptides were separated on a 125-mm C18 reversed-phase column with an inner diameter of 100 µm (Nikkyo Technos) with a linear 5%–40% acetonitrile gradient for 0–100 min, followed by an increase to 95% acetonitrile for 5 min. The mass spectrometer was operated in DIA mode. The MS1 spectra were collected in the range of m/z 500–740 (isolation window width, 10 Da) at 70 000 resolution to set an automatic gain control (AGC) target of 3 × 10^6^. MS2 spectra were collected at 200–1800 m/z at 70 000 resolution to set an AGC of 3 × 10^6^, a maximum injection time of “auto,” and stepped normalized collision energies of 22%. All DIA raw data were processed with DIA-NN (version 1.8.1) in the library-free search mode with reference to human or mouse UniProt sequences.

### Pulse-SILAC protein turnover analysis

Cells were seeded in poly-L-lysine-coated six-well plates at 2 × 10^5^ cells per well and cultured overnight in high-glucose SILAC DMEM supplemented with 10% dialyzed FBS, penicillin–streptomycin, L-leucine, L-methionine, and unlabeled L-arginine and L-lysine. For pulse labeling, the medium was replaced with heavy SILAC medium containing L-arginine-U-^13^C_6_,^15^N_4_ and L-lysine-U-^13^C_6_,^15^N_2_. Cells were harvested at 0, 3, 6, 9, 12, and 24 h after medium replacement. Total proteins were extracted using a lysis buffer [50 mM Tris–HCl (pH 8.8) and 1% SDS]. Protein concentration of the lysate was adjusted to 1 mg/ml, and cysteine residues were reduced with 2.5 mM tris(2-carboxyethyl)phosphine hydrochloride (Thermo Fisher Scientific) for 30 min at 37 °C, and then alkylated with 5 mM 2-iodoacetamide (Sigma–Aldrich) for 30 min at room temperature. Proteins were precipitated by adding NaCl to a final concentration of 30 mM, followed by the addition of three volumes of cold acetone. After centrifugation, the resulting protein pellets were washed with 90% acetone, resuspended in 100 mM HEPES (pH 8.0) by sonication, and digested with trypsin (Roche) for 16 h. After centrifugation, the supernatants corresponding to 1 μg of protein were purified using Evotip Pure (Evosep, Denmark) according to the manufacturer’s instructions. Peptides were separated on an EV1137 column (Evosep, Denmark) using a pre-programmed gradient for 30 samples per day on the Evosep One system (Evosep, Denmark). Eluted peptides were electrosprayed using a LOTUS emitter ([20]-05, FOSSILIONTECH, Spain) coupled to an Orbitrap Exploris 480 mass spectrometer (Thermo Fisher Scientific). The instrument was operated in DIA mode with positive ion polarity, and all spectra were acquired in profile mode. Spray voltage was set to 2.2 kV, funnel RF level to 40, and heated capillary temperature to 275°C. For MS1 acquisition, the resolution was set to 120 000 at m/z 200 with an AGC target of 100% and a maximum injection time of 45 ms over an m/z range of 400–1200. For MS2 acquisition, the resolution was set to 30 000 with auto injection time and an m/z range of 145–1450. The AGC target for fragment ion spectra was set to 1000%. Twenty-seven DIA windows of 6 m/z with 1 m/z overlap were used across the range m/z 479.5–885.5. Normalized collision energy was set to 28%. Samples cultured in unlabeled amino acid-containing medium (time 0) were initially analyzed using DIA-NN in library-free search mode against the human UniProt database (accessed 27 April 2021). Enzyme specificity was set to “Trypsin/P” with up to one missed cleavage allowed. Carbamidomethylation of cysteine was specified as a fixed modification, whereas protein N-terminal acetylation was included as a variable modification. Based on the identified precursor ions, an *in silico* precursor library for labeled peptides was generated by applying mass shifts of +8.014199 Da for lysine residues and +10.008269 Da for arginine residues. The unlabeled and labeled precursor libraries were subsequently merged, and all files were searched against the combined library. Pulsed-SILAC data were analyzed using a modified protein turnover model based on the method described by Schwanhäusser *et al*. [43]. For each precursor, heavy-to-light SILAC ratios (H/L) were calculated at each time point. Replicate values were filtered using the Grubbs’ outlier test (α = 0.01), and the remaining values were summarized as medians for each time point. Precursors showing H/L ratios >0.1 at time 0 were excluded from further analysis. Protein turnover was modeled with correction for cell doubling time. Degradation rate constants (${{k}_{\mathrm{ deg}}}$) were estimated from the following linearized relationship:


\begin{eqnarray*}
{{k}_{\mathrm{ deg}}} = \frac{{\mathop \sum \nolimits_i {{t}_i}{\mathrm{ln}}\left( {{{r}_i} + 1} \right)}}{{\mathop \sum \nolimits_i t_i^2}} - \frac{{{\mathrm{ln}}2}}{{{{t}_{cc}}}},
\end{eqnarray*}

where ${{r}_i}$ represents the heavy-to-light SILAC ratio at time point ${{t}_i}$, and ${{t}_{cc}}$denotes the cell doubling time. To identify the optimal fitting interval, regression analysis was performed using all available time points as well as sequentially truncated subsets from both the beginning and end of the time series, provided that at least three time points were included. The subset yielding the highest Pearson correlation coefficient was selected for half-life estimation. Protein half-lives were calculated as


\begin{eqnarray*}
{{T}_{1/2}} = \frac{{\ln 2}}{{{{k}_{\mathrm{ deg}}}}}.
\end{eqnarray*}

Protein-level half-lives were determined by taking the median of precursor half-lives assigned to each protein.

### Statistical analysis

The generalized linear model in RiboDiff was used to analyze the differential translation efficiency (TE) [44]. The effect size was calculated using Cliff’s delta. Two-sided Student’s *t*-test and Mann–Whitney *U* tests were performed using Scipy (v1.2.1) in Python 3.6.8. An adjusted *P*-value (false discovery rate *q* value < 0.05) was used for the extraction of differentially expressed genes. *P*-values < .05 were otherwise considered statistically significant. All experiments were performed at least twice and yielded similar results.

## Results

### Impact of RPL41 deficiency on polysome formation, protein synthesis, and cell proliferation

**Figure 1.**
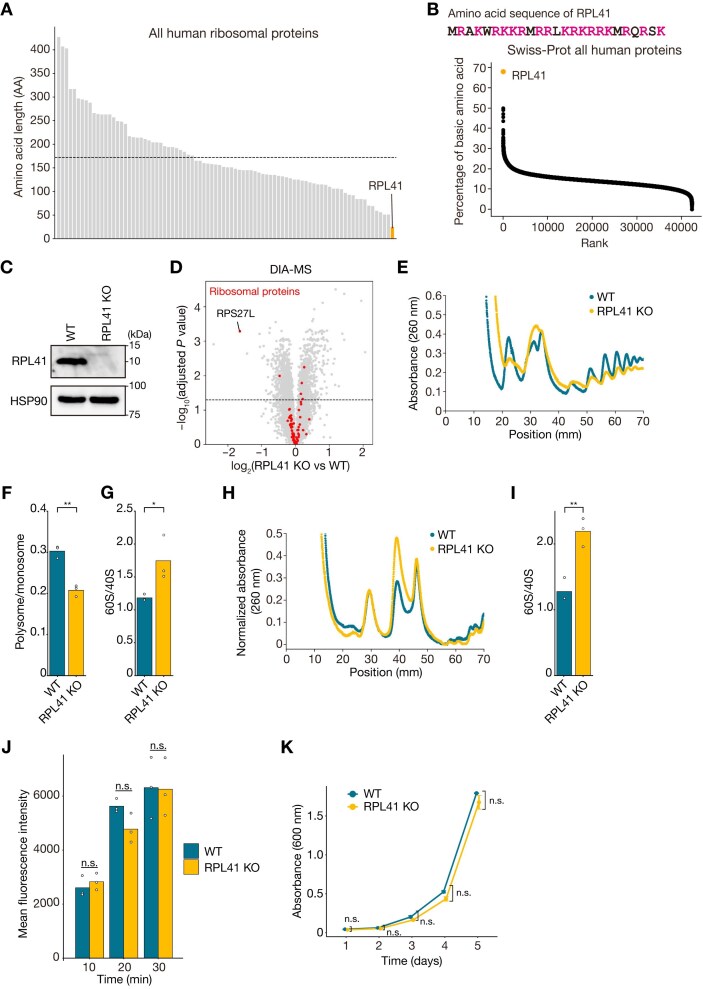
Characterization of RPL41 deficiency in human cells. (**A**) Amino acid lengths of human RPs. RPL41 is the shortest among all RPs. The dashed line indicates the average length of all RPs. (**B**) Proportion of basic amino acids (lysine and arginine) in all human proteins. RPL41 shows the highest proportion. The amino acid sequence of RPL41 is shown, with basic residues highlighted in red. (**C**) Immunoblot analysis of RPL41 KO and WT HEK293T cells using the indicated antibodies. HSP90 was used as a loading control. (**D**) Volcano plot of protein abundance measured using data-independent acquisition mass spectrometry (DIA-MS) in WT and RPL41 KO cells (*n* = 4). RPs are highlighted in red. *P*-values were calculated using a two-tailed Student’s *t*-test and adjusted for multiple comparisons using the Benjamini–Hochberg method. The horizontal dashed line indicates an adjusted *P*-value of 0.05. (**E**) Polysome profiling of WT and RPL41 KO cells via sucrose density gradient centrifugation. Quantification of the ratio of polysomes to monosomes (**F**) and the ratio of 60S to 40S subunits (**G**) (*n* = 3). Polysome profiling of WT and RPL41 KO cells via sucrose density gradient centrifugation using the extended centrifugation protocol (**H**), and quantification of the ratio of 60S to 40S subunits (**I**) (*n* = 3). (**J**) Flow cytometry analysis of O-propargyl-puromycin (OP-puro) incorporation at indicated times (*n* = 3). Data represent the mean fluorescence intensity in WT and RPL41 KO cells. (**K**) Cell proliferation assay of WT and RPL41 KO cells (*n* = 3). *P < .05, **P < .01, ***P < .001; n.s., not significant [two-sided Student’s *t*-test (F, G, and I), two-sided Welch’s *t*-test (J), and two-way analysis of variance (ANOVA), followed by Sidak’s multiple-comparison test (K)].

RPL41 is the smallest human ribosomal protein and is distinguished by its unusually high proportion of basic amino acids (Fig. 1A and B). Given the limited understanding of RPL41 function in mammalian systems, we generated RPL41-deficient HEK293T cells using CRISPR–Cas9-mediated genome editing. Insertion-deletion mutations at the RPL41 locus resulted in complete loss of RPL41 protein expression (Fig. 1C). To determine whether RPL41 deficiency alters ribosomal composition, we performed quantitative proteomic profiling using DIA-MS. Although the abundance of most RPs remained unchanged, a subset showed modest alterations, including a pronounced reduction in RPS27L expression (Fig. 1D and Supplementary Table S3). As expected, RPL41 itself could not be detected by MS owing to its small size and enrichment in lysine and arginine residues, even in WT cells.

Next, we examined polysome profiles by sucrose density gradient centrifugation. Using the 2-h centrifugation protocol, RPL41 KO cells exhibited a reduction in polysome abundance relative to monosomes (Fig. 1E and F). Under these conditions, the distribution of ribosomal subunits was altered, with an apparent increase in free 60S subunits and a decrease in free 40S subunits (Fig. 1E and G). To resolve these subunits more clearly, we repeated the analysis using an extended centrifugation protocol, which improved separation of the 40S, 60S, and 80S peaks. Under these conditions, the 60S/40S ratio was significantly increased in RPL41 KO cells compared with WT cells (Fig. 1H and I), confirming that loss of RPL41 alters the steady-state balance of ribosomal species.

We then asked whether these changes were accompanied by a reduction in global protein synthesis. OP-puro incorporation measured by flow cytometry did not differ significantly between WT and RPL41 KO cells at 10, 20, or 30 min of labeling (Fig. 1J), indicating that bulk protein synthesis was not detectably reduced under these conditions despite the altered ribosome profile.

Finally, we assessed whether these changes influenced cell growth. RPL41 KO cells showed proliferation rates comparable to those of WT cells over the time course examined (Fig. 1K). Taken together, these findings indicate that RPL41 deficiency alters polysome organization and ribosomal subunit balance without causing a detectable decrease in bulk protein synthesis or a significant defect in cell proliferation under these conditions.

### Structural analysis of RPL41 using cryo-electron microscopy

RPL41 forms the eukaryote-specific intersubunit bridge eB14 located at the center of the ribosome, where it functions as an axis for rotation between the 40S body and the platform [22, 23, 37]. This bridge is flanked by other intersubunit bridges (B2a, B2b, B2c, and B3), which are composed of evolutionarily conserved rRNA elements also present in bacteria. In eukaryotic ribosomes, RPL41 establishes additional contacts with RPL19/eL19 and RPL24/eL24, thereby contributing to the subunit association and ribosome stability [25]. Although structural insights into RPL41 have accumulated, its direct role in intersubunit dynamics remains poorly understood. Therefore, we analyzed ribosomes with and without RPL41 using single-particle cryo-EM.

**Figure 2.**
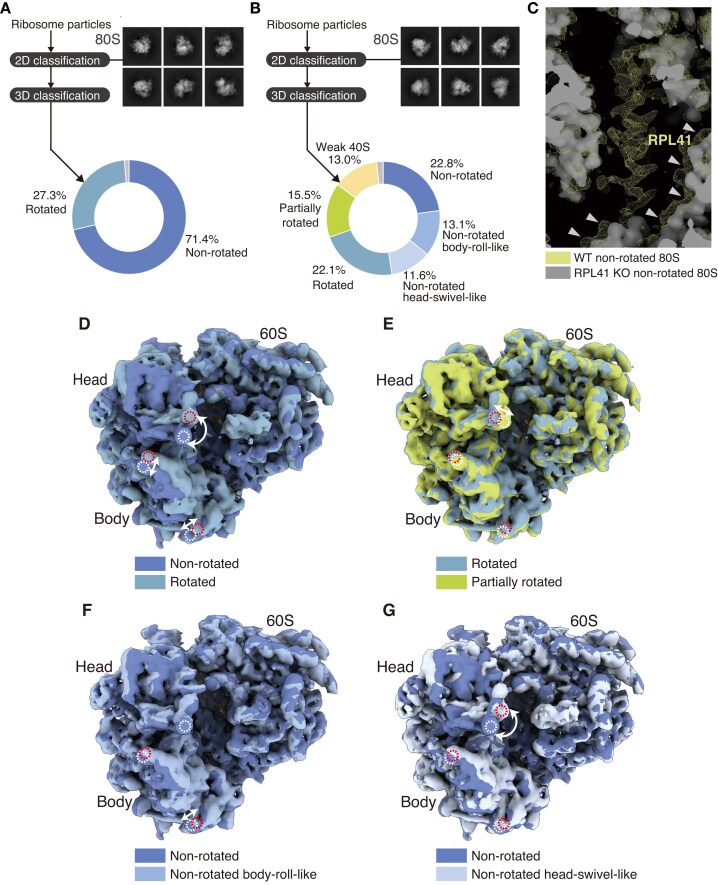
Cryo-EM analysis reveals increased conformational heterogeneity of RPL41-deficient 80S ribosomes. Cryo-EM classification workflows of 80S ribosomes purified from WT (**A**) and RPL41 KO (**B**) HEK293T cells. Representative 2D class averages and 3D class distributions are shown. WT ribosomes were classified mainly into non-rotated and rotated 80S states, whereas RPL41-deficient ribosomes were distributed across multiple 80S conformations, including non-rotated, rotated, partially rotated, non-rotated body-roll-like, non-rotated head-swivel-like, and weak-40S classes. (**C**) Close-up view of the intersubunit interface around the RPL41/eB14 region in non-rotated WT and RPL41-deficient 80S ribosomes. RPL41 density is absent in the KO map. Arrowheads indicate the region showing subtle local widening or weakening of the intersubunit interface near the bridge region. Overlays of representative RPL41 KO 80S subclasses from the 3D classification after alignment on the 60S subunit. Shown are comparisons between non-rotated and rotated classes (**D**), rotated and partially rotated classes (**E**), non-rotated and non-rotated body-roll-like classes (**F**), and non-rotated and non-rotated head-swivel-like classes (**G**). Dotted circles indicate representative regions with visible displacement of 40S density, and arrows indicate the direction of displacement. These overlays illustrate the diverse positioning of the 40S subunit in RPL41-deficient ribosomes.

80S ribosomes purified from WT or RPL41 KO HEK293T cells were subjected to cryo-EM. In the WT dataset, 2D classification revealed a homogeneous population of intact 80S particles (Fig. 2A and Supplementary Fig. S1). Subsequent 3D classification showed that most ribosomes adopted a non-rotated conformation, whereas a smaller fraction displayed a rotated state (Fig. 2A and Supplementary Fig. S1). In the RPL41-deficient dataset, 2D classification also recovered intact 80S particles (Fig. 2B and Supplementary Fig. S2). However, 3D classification revealed a more heterogeneous distribution of 80S conformations, including non-rotated, rotated, partially rotated, non-rotated body-roll-like, non-rotated head-swivel-like, and weak-40S classes (Fig. 2B and Supplementary Fig. S2). These results indicate that loss of RPL41 does not cause gross disruption of 80S ribosome architecture but instead increases conformational heterogeneity of the 80S ribosome, particularly in the relative positioning of the 40S subunit.

A direct comparison of non-rotated WT and RPL41-deficient 80S maps confirmed the absence of density corresponding to RPL41 in the KO ribosome (Fig. 2C). This comparison did not reveal a major global rearrangement of the averaged 80S structure. However, a subtle local widening of the intersubunit interface was observed near the decoding-center-proximal bridge region, including the region around bridge B2a. These observations suggest that loss of RPL41 locally weakens or loosens the intersubunit interface without causing complete dissociation of the 40S and 60S subunits.

To further visualize the conformational diversity of RPL41-deficient ribosomes, we compared representative 80S maps from the 3D classification after alignment on the 60S subunit. In the RPL41-deficient dataset, the non-rotated and rotated classes showed the expected relative displacement of the 40S subunit, similar to the corresponding conformational states observed in the WT dataset (Fig. 2D). In addition to these canonical conformations, 3D classification identified a partially rotated class, which showed a more pronounced deviation from the rotated class and indicated that RPL41-deficient ribosomes populate additional 40S orientations beyond the canonical non-rotated and rotated states (Fig. 2E). Other non-rotated classes showed altered positioning of the 40S body or head region, suggestive of body-roll-like and head-swivel-like conformations (Fig. 2F and G). Because these purified ribosomes showed little detectable mRNA or transfer RNA (tRNA) density, we do not interpret these classes as defined translocation intermediates. Rather, they indicate that RPL41-deficient 80S ribosomes sample a broader range of conformational states even in the absence of defined elongation ligands.

Consistent with previous structural studies of eukaryotic ribosomes [22, 23, 37], RPL41 is positioned in a socket-like environment formed by rRNA helices near the subunit interface. In the WT non-rotated ribosome, the α-helix of RPL41 is located between helices 44 and 45 of the 18S rRNA, where its basic residues contact the phosphate backbone near the decoding center (Supplementary Fig. S3A). Additional contacts with helix 27 of the 18S rRNA and the 28S rRNA contribute to the eukaryote-specific eB14 bridge (Supplementary Fig. S3B and C). Comparison of non-rotated and rotated ribosomes further showed that RPL41 remains positioned at the intersubunit interface across these conformational states (Supplementary Fig. S3D). Thus, although RPL41 is not required for the gross architecture of the 80S ribosome, it appears to constrain the conformational landscape of the ribosome by stabilizing the relative positioning of the 40S subunit at the intersubunit interface.

### RPL41 loss perturbs A-site dynamics and slows elongation

**Figure 3.**
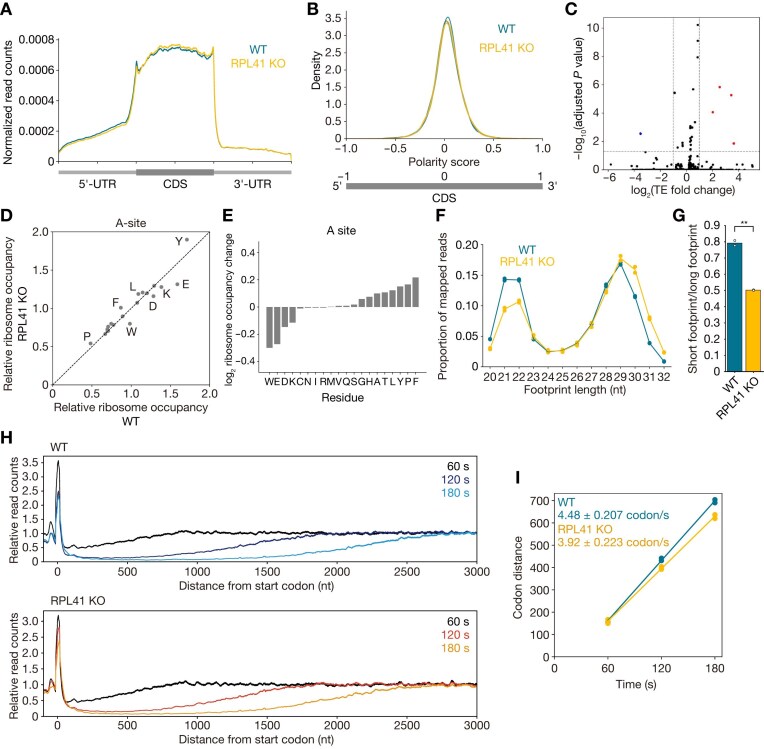
Reduced translation elongation speed in cells lacking RPL41. (**A**) Metagene plots of ribosome footprints obtained using Ribo-seq from WT and RPL41 KO cells (*n* = 2). Data represent the average of two replicates. (**B**) Distributions of polarity scores for 31 862 transcripts from WT and RPL41 KO cells are plotted (top panel). Schematic representation of polarity score (bottom panel). (**C**) Volcano plot of differential TE determined using Ribo-seq and RNA-seq analyses (*n* = 2). Changes in TE for each transcript in RPL41 KO versus WT cells were analyzed using RiboDiff. *P*-values were calculated using the chi-square test and adjusted for multiple comparisons using the Benjamini–Hochberg method. Vertical and horizontal dashed lines indicate log_2_ fold changes of ±1 and an adjusted *P*-value of .05, respectively. (**D**) Relative ribosome occupancy at A-site codons in WT and RPL41 KO cells (*n* = 2), aggregated across all codons encoding each amino acid. (**E**) Fold changes in ribosome occupancy at A-site codons for each amino acid residue in RPL41 KO relative to WT cells (*n* = 2). Distribution of ribosome-protected fragment (RPF) lengths in WT and RPL41 KO cells (**F**), and quantification of the ratio of short footprint (20–22 nt) to long footprint (28–30 nt) (**G**) (*n* = 2). **P < .01 (two-tailed Student’s *t*-test). (**H**) Metagene profiles of ribosome run-off assays with harringtonine chase in WT and RPL41 KO cells (*n* = 2), showing relative read density from the start codon to 3000 nt. Values represent the average of biological replicates (*n* = 2). (**I**) Estimation of translation elongation rates using linear regression analysis with 95% confidence intervals.

Given that cryo-EM analysis indicated increased conformational heterogeneity of RPL41-deficient ribosomes, particularly in the relative positioning of the 40S subunit, we next asked whether this structural phenotype is associated with altered translational dynamics in cells [45, 46]. In all samples, most ribosome footprints mapped to coding sequences (CDSs), biological replicates were highly reproducible, and the libraries displayed clear three-nucleotide periodicity, confirming the high quality of the libraries (Supplementary Fig. S4A and B, and Supplementary Table S4) [47]. Metagene analysis showed no obvious changes in footprint distribution across the 5′-untranslated region (UTR), CDS, or 3′-UTR upon RPL41 loss (Fig. 3A). To quantify ribosome positioning more precisely, we calculated polarity scores based on ribosome footprint distributions along individual transcripts, where values approaching −1 indicate enrichment at the 5′ end and those near +1 reflect enrichment at the 3′ end [48]. Polarity scores did not differ between WT and RPL41 KO cells, providing no evidence for widespread premature ribosome dissociation during translation (Fig. 3B and Supplementary Table S5).

We then assessed TE (defined as Ribo-seq reads normalized to RNA-seq reads) and found that only a small number of transcripts displayed significant changes upon loss of RPL41 (Fig. 3C and Supplementary Table S6). At the codon level, analysis of A-site ribosome occupancy revealed modest but non-unidirectional changes in RPL41 KO cells. Several codons, including those encoding glutamic acid (E), lysine (K), aspartic acid (D), and tryptophan (W), showed decreased relative occupancy, whereas some other codons showed increased occupancy (Fig. 3D and E). This decrease was further supported by RUST normalization, which also showed reduced ribosome occupancy at these codons in RPL41 KO cells (Supplementary Fig. S4C) [40].

Additionally, the distribution of RPF lengths was altered in RPL41 KO cells (Fig. 3F). Shorter footprints (20–22 nt) are typically associated with ribosomes bearing an open A-site, whereas longer footprints (28–30 nt) correspond to ribosomes with tRNA-occupied A sites [49, 50]. Consistent with this, the ratio of short to long footprints was significantly reduced in RPL41-deficient cells (Fig. 3G), suggesting altered A-site dynamics and a relative shift in ribosome states.

We next performed ribosome run-off assays to measure the elongation speed [51]. Cells were treated with harringtonine, which inhibits the first elongation step at the start codon, thereby preventing new initiation events. Subsequently, translation elongation was arrested at the defined time points by adding CHX. Ribosome profiling under these conditions revealed a time-dependent expansion of ribosome-free regions downstream of the start codons, reflecting ribosome progression along the mRNAs (Fig. 3H). Regression analysis of these ribosome-free zones indicated that elongation was slower in RPL41 KO cells than in controls, with estimated rates of 4.48 ± 0.21 and 3.92 ± 0.22 codons per second in WT and RPL41 KO cells, respectively (95% confidence intervals; Fig. 3I). For example, the synthesis of a 1000-residue protein was predicted to take ~32 s longer in RPL41 KO cells. These findings suggest that the loss of RPL41 in ribosomes perturbs A-site dynamics and slows translational elongation.

### Effects of RPL41 deficiency on ribosome collisions in HEK293T cells

Premature stalling of ribosomes during translation can lead to the accumulation of potentially toxic, incomplete nascent protein chains. These aberrant products are normally eliminated by the ribosome-associated quality control (RQC) pathway, which detects stalled ribosomes and targets nascent peptides for degradation [52, 53]. When a leading ribosome stalls, trailing ribosomes may collide with it, generating diribosomes (disomes) that are recognized by the RQC machinery for dissociation from mRNA and clearance of the stalled chains.

**Figure 4.**
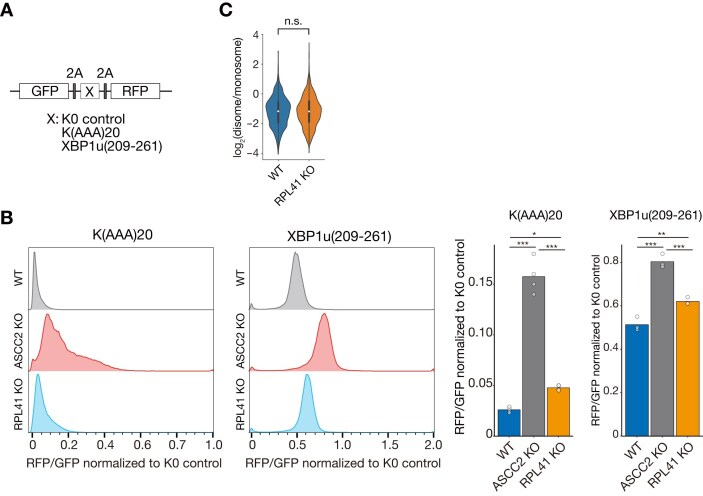
Reporter- and disome-seq–based analysis of ribosome collisions. (**A**) Schematic of the dual-fluorescence reporter used to measure readthrough of collision-induced stalling sequences. GFP and RFP were connected by either a non-stalling control sequence (K0), a stalling sequence of 20 consecutive AAA codons (K(AAA)20), or a stalling region of XBP1u (amino acids 209–261). Each sequence was flanked by 2A peptide elements, ensuring independent translation of GFP and RFP. (**B**) Histograms (left) and quantification (right) of RFP/GFP ratios measured using flow cytometry in WT, ASCC2 KO, and RPL41 KO cells stably expressing indicated reporters [*n *= 4 for K(AAA)20; 3 for XBP1u(209–261)]. Values are normalized to the K0 control reporter. (**C**) Disome intensity normalized by monosome intensity of WT and RPL41 KO cells (*n* = 2). *P < .05, **P < .01, ***P < .001; n.s., not significant [one-way ANOVA, followed by Tukey’s test (**B**) or two-tailed Mann–Whitney *U* test (**C**)].

Given that RPL41-deficient cells exhibit reduced translation elongation rates, we examined whether loss of RPL41 influences ribosome collisions and their resolution. Disomes are normally disassembled by the hRQT complex (ASC-1 complex), which facilitates their removal from mRNA [54–57]. To evaluate the handling of collision-inducing sequences, we used dual-fluorescence reporters containing either 20 consecutive AAA lysine codons [K(AAA)20] or amino acids 209–261 of XBP1u, together with a non-stalling control (K0). The readthrough efficiency was quantified as the ratio of downstream red fluorescent protein (RFP) expression to upstream GFP expression (Fig. 4A). As expected, ASCC2 depletion markedly increased readthrough, consistent with its established role in removing collided ribosomes. Under the same conditions, RPL41 deficiency modestly increased readthrough, although to a lesser extent than ASCC2 depletion (Fig. 4B). To test whether this modest effect could be explained indirectly by altered translation of RQC factors, we examined both RPF abundance and TE values for 24 RQC-related genes. The RQC-related gene set showed no significant collective decrease in RPF abundance in RPL41 KO cells compared with all detected transcripts (Supplementary Fig. S5A and Supplementary Table S7). TE values for the same gene set also showed no significant collective shift (Supplementary Fig. S5B and Supplementary Table S7). These results argue against an indirect explanation through reduced translation of RQC components.

To further assess ribosomal collisions, we performed disome profiling (disome-seq) [58–60]. RPL41 deficiency did not lead to a significant global change in the disome/monosome ratio (Fig. 4C and Supplementary Table S8). In addition, inspection of the endogenous *XBP1u* locus did not reveal an obvious difference in monosome or disome peaks between WT and RPL41 KO cells (Supplementary Fig. S5C). These results indicate that, in HEK293T cells, loss of RPL41 produces at most a subtle effect on the handling of selected collision-inducing sequences, which is detectable in the reporter assay but not robustly resolved by global disome profiling.

### Altered translational fidelity in RPL41-deficient cells

**Figure 5.**
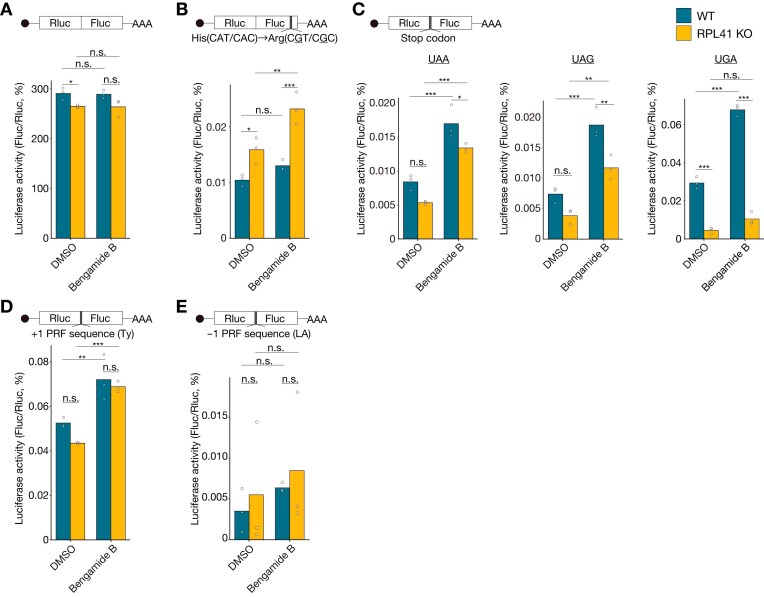
Translational fidelity analysis using tandem Rluc–Fluc reporters. Each panel shows a schematic of the reporter construct (top) and the corresponding experimental results (bottom). Control reporter in which Rluc and Fluc are linked by a 9-amino acid sequence (**A**). Reporter for detecting amino acid misincorporation, carrying a point mutation in the Fluc active site (**B**). Reporter containing a stop codon within the linker sequence to assess stop codon readthrough (**C**). Reporters containing −1 or +1 PRF sequences to evaluate frameshifting efficiency (**D, E**). WT and RPL41 KO cells were analyzed under dimethyl sulfoxide control or bengamide B treatment conditions across all reporter assays (*n* = 3). *P < .05, **P < .01, ***P < .001; n.s., not significant (two-way ANOVA, followed by Sidak’s multiple-comparison test).

In yeast, loss of RPL41 has been reported to compromise translational fidelity, leading to an increased frequency of −1 ribosomal frameshifting events [27]. To determine whether RPL41 deficiency exerts a similar effect in mammalian cells, we employed a dual-luciferase reporter system in HEK293T cells coexpressing Rluc and Fluc. In this assay, the control reporters expressed Rluc and Fluc joined by a nine–amino acid linker, whereas the misincorporation reporter contained a point mutation in the Fluc active site, rendering Fluc activity detectable only when amino acid misincorporation occurred during translation (Fig. 5A and B) [61]. To assess stop codon readthrough, we used a reporter carrying a stop codon between Rluc and Fluc, where increased Fluc luminescence reflected the readthrough (Fig. 5C). Similarly, programmed ribosomal frameshifting (PRF) was evaluated using reporters containing −1 or +1 PRF sequences, with elevated Fluc luminescence indicating increased frameshifting (Fig. 5D and E).

In mammalian cells, the inhibition of methionine aminopeptidase by bengamide B prevents the removal of the N-terminal initiator methionine from RPs, thereby perturbing the ribosome structure and increasing translation errors [62, 63]. Consistent with previous reports, bengamide B treatment of HEK293T cells enhanced stop codon readthrough (Fig. 5C). In RPL41-deficient cells, misincorporation rates significantly increased even under basal conditions and were further exacerbated by bengamide B treatment (Fig. 5A and B). Stop codon readthrough was most strongly reduced at the UGA codon in RPL41-deficient cells, whereas UAA and UAG readthrough were significantly decreased only in the presence of bengamide B (Fig. 5C). No significant changes in −1 or +1 PRF frequencies were observed under any condition (Fig. 5D and E). These results demonstrate that the loss of RPL41 in mammalian cells increases amino acid misincorporation but enhances the accuracy of stop codon recognition without affecting PRF.

### Impact of RPL41 loss on the expression of long proteins

**Figure 6.**
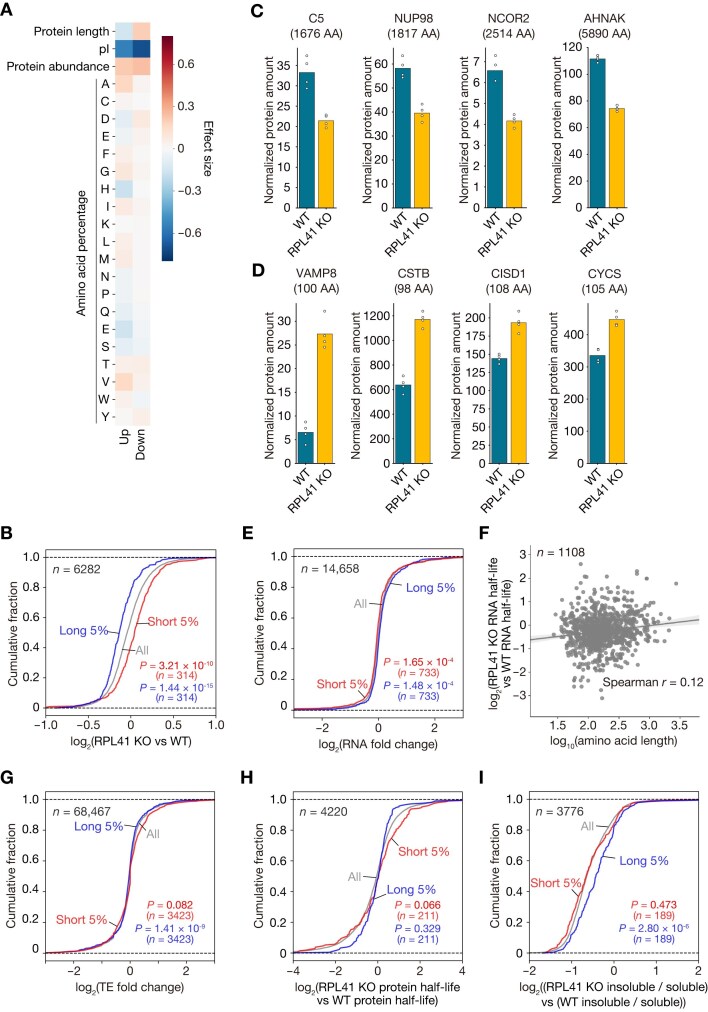
Loss of RPL41 alters protein homeostasis with preferential reduction of long proteins. (**A**) Effect sizes (Cliff’s delta, *d*) for selected protein features, including length, isoelectric point (pI), abundance, and amino acid composition in RPL41 KO versus WT cells. (**B**) Cumulative distributions of fold changes in protein abundance between RPL41 KO and WT cells (*n* = 4), based on DIA-MS data. Shown are all detected proteins (*n* = 6282), the top 5% longest proteins (*n* = 314), and the bottom 5% shortest proteins (*n* = 314). *P*-values were calculated using a two-tailed Mann–Whitney *U* test. Abundance of representative long proteins (C5, NUP98, NCOR2, and AHNAK) (**C**) and short proteins (VAMP8, CSTB, CISD1, and CYCS) (**D**) in WT and RPL41 KO cells as measured using DIA-MS. The amino acid length of each protein is indicated. (**E**) Cumulative distributions of fold changes in mRNA abundance between RPL41 KO and WT cells (*n* = 3). Shown are all detected genes (*n* = 14, 658), those encoding the top 5% longest proteins (*n* = 733), and those encoding the bottom 5% shortest proteins (*n* = 733). The isoform with the longest amino acid chain was selected. *P*-values were calculated using a two-tailed Mann–Whitney *U* test. (**F**) Correlation between fold change in half-life of transcripts measured using SLAM-seq in WT and RPL41 KO cells and amino acid length. Coding transcripts detected in both WT and RPL41 KO were plotted (*n* = 1108). (**G**) Cumulative distributions of fold changes in TE between RPL41 KO and WT cells (*n* = 2). Shown are all detected transcripts (*n* = 68, 467), transcripts encoding the top 5% longest proteins (*n* = 3423), and transcripts encoding the bottom 5% shortest proteins (*n* = 3423). *P*-values were calculated using a two-tailed Mann–Whitney *U* test. (**H**) Cumulative distributions of fold changes in protein half-life measured by pulse-SILAC between RPL41 KO and WT cells (*n* = 3). Shown are all detected proteins (*n* = 4220), the top 5% longest proteins (*n* = 211), and the bottom 5% shortest proteins (*n* = 211). *P-*values were calculated using a two-tailed Mann–Whitney *U* test. (**I**) Cumulative distributions of fold changes in insoluble protein abundance normalized to the soluble fraction in RPL41 KO versus WT cells (*n* = 3). Shown are all detected proteins (*n* = 3776), the top 5% longest proteins (*n* = 189), and the bottom 5% shortest proteins (*n* = 189). *P-*values were calculated using a two-tailed Mann–Whitney *U* test.

To investigate how altered translation dynamics caused by RPL41 deficiency affect protein expression, we performed an in-depth analysis of the DIA-MS data. Compared with control cells, RPL41-deficient cells exhibited increased expression of 621 proteins and decreased expression of 790 proteins (Fig. 1D and Supplementary Table S3). To characterize these differentially expressed proteins, we examined their expression changes in relation to attributes such as protein length, isoelectric point, baseline expression levels in WT HEK293T cells, and amino acid composition. Among these factors, protein length showed a clear correlation with changes in expression; proteins upregulated in RPL41-deficient cells tended to be shorter, whereas those downregulated tended to be longer (Fig. 6A).

A comparison of the top 5% of the longest proteins with the bottom 5% of the shortest proteins confirmed this trend, revealing significantly reduced expression of the longest proteins and significantly increased expression of the shortest proteins (Fig. 6B). Representative examples of long proteins with reduced abundance included C5 (1676 amino acids), NUP98 (1817 amino acids), NCOR2 (2514 amino acids), and AHNAK (5890 amino acids), whereas representative short proteins with increased abundance included VAMP8 (100 amino acids), CSTB (98 amino acids), CISD1 (108 amino acids), and CYCS (105 amino acids) (Fig. 6C and D).

First, to test whether the reduced expression of long proteins might be attributable to decreased mRNA levels, we compared the abundance of their transcripts between control and RPL41-deficient cells using RNA-seq. No consistent decrease in the transcript levels of genes encoding long proteins was observed (Fig. 6E and Supplementary Table S9). Next, we assessed mRNA stability using Thiol(SH)-Linked Alkylation for the Metabolic Sequencing of RNA (SLAM-seq), which involves pulse-labeling of nascent transcripts with 4-thiouridine and calculating half-lives from the decay of labeled RNA fractions. This analysis likewise revealed no evidence that transcripts encoding long proteins were selectively destabilized in RPL41-deficient cells (Fig. 6F and Supplementary Table S10).

Changes in TE and RPF abundance for transcripts encoding long proteins were statistically significant but quantitatively modest and were insufficient to explain the pronounced reduction in long-protein abundance (Fig. 6G, Supplementary Fig. S6A, and Supplementary Table S11). We therefore tested whether the reduced abundance of long proteins might reflect enhanced ribosome drop-off during translation. We stratified transcripts by encoded protein length and reanalyzed polarity scores. However, no significant difference between WT and RPL41 KO cells was detected in any length group, arguing against a length-dependent ribosome drop-off model (Supplementary Fig. S6B).

We next asked whether the selective depletion of long proteins might instead reflect enhanced protein turnover. However, pulse-SILAC analysis showed that the half-lives of long proteins were not significantly reduced in RPL41-deficient cells (Fig. 6H and Supplementary Table S12), arguing against accelerated degradation as the primary explanation. We also examined the insoluble protein fraction and found that long proteins showed a significant increase in the insoluble-to-soluble ratio in the absence of RPL41 (Fig. 6I and Supplementary Table S13). This preferential partitioning of long proteins into the insoluble fraction suggests that their reduced abundance in the soluble proteome may be associated, at least in part, with impaired folding or proteostasis rather than enhanced degradation.

To further determine whether the proteins reduced in the DIA-MS dataset could be explained by transcriptional, translational, or post-translational changes, we focused on proteins whose abundance was decreased in RPL41 KO cells. The corresponding transcripts showed statistically significant but quantitatively modest decreases in RNA and RPF abundance, whereas their TE values did not exhibit a significant collective shift (Supplementary Fig. S6C–H). Protein half-life also showed only a small overall shift in this MS-down group (Supplementary Fig. S6I). At the individual-protein level, however, the relative contributions of RNA abundance, ribosome occupancy, and protein stability varied substantially, indicating that the proteins reduced in the DIA-MS dataset comprise mechanistically heterogeneous subsets.

Together, these results indicate that the preferential reduction of long proteins cannot be attributed solely to changes in RNA abundance, ribosome occupancy, length-dependent ribosome drop-off, or protein half-life. Rather, the increased insolubility of long proteins, together with modest reductions in their translational output, suggests that subtle defects in elongation and translational fidelity caused by RPL41 loss cumulatively impair long-protein biogenesis and proteostasis.

### Developmental and growth defects associated with Rpl41 KO in mice

**Figure 7.**
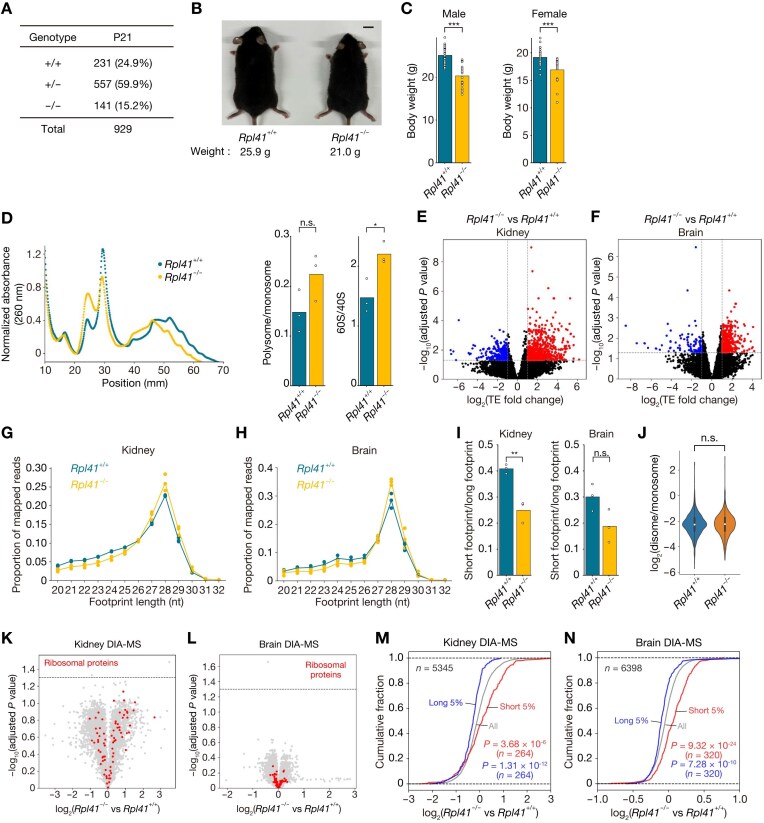
Physiological and translational impact of RPL41 deficiency in mice. (**A**) Genotypic distribution and percentages of offspring at weaning from heterozygous (*Rpl41*^+/−^) intercrosses. (**B**) Representative images of *Rpl41*^+/+^ and *Rpl41*^−/−^ mice with corresponding body weights indicated. Scale bar, 1 cm. (**C**) Body weights of *Rpl41*^+/+^ (*n *= 44 for male; 31 for female) and *Rpl41*^−/−^ (*n *= 23 for male; 18 for female) mice at 8 weeks of age. ***P < .001 (two-tailed Student’s *t*-test). (**D**) Polysome profiling of kidney lysates from *Rpl41*^+/+^ and *Rpl41*^−/−^ mice via sucrose density gradient centrifugation, with quantification of polysome-to-monosome and 60S-to-40S ratios (*n* = 3, male). *P < .05; n.s., not significant (two-tailed Student’s *t*-test). Volcano plots of differential TE determined using Ribo-seq and RNA-seq in the kidney (**E**) and brain (**F**) of *Rpl41*^−/−^ versus *Rpl41*^+/+^ mice (*n* = 3, male). TE changes were calculated using RiboDiff. *P*-values were derived from chi-square tests and adjusted for multiple comparisons using the Benjamini–Hochberg method. The dashed line indicates an adjusted *P*-value of 0.05. Distribution of RPF lengths in the kidney (**G**) and brain (**H**) of *Rpl41*^+/+^ and *Rpl41*^−/−^ mice (*n* = 3, male), and quantification of the ratio of short footprint (20–22 nt) to long footprint (28–30 nt) (**I**) (*n* = 3). **P < .01; n.s., not significant (two-tailed Student’s *t*-test). (**J**) Disome intensity normalized by monosome intensity of *Rpl41*^+/+^ and *Rpl41*^−/−^ mouse kidneys (*n* = 3, male). (K and L) Volcano plots of protein abundance measured using DIA-MS in the kidney (**K**) and brain (**L**) of *Rpl41*^+/+^ and *Rpl41*^−/−^ mice (*n *= 3 for the male kidney; 4 for the male brain). RPs are shown in red. *P*-values were calculated using Student’s *t*-tests and adjusted for multiple comparisons using the Benjamini–Hochberg method. Vertical and horizontal dashed lines indicate log_2_ fold changes of ±1 and an adjusted *P*-value of .05, respectively. Cumulative distributions of fold changes in protein abundance in the kidney (**M**) and brain (**N**) of *Rpl41*^−/−^ mice relative to *Rpl41*^+/+^ mice (*n *= 3 for the male kidney; 4 for the male brain), determined using DIA-MS. Data are shown for all proteins (*n* = 5, 345 for the male kidney; 6, 398 for the male brain), the top 5% longest proteins (*n* = 264 for the male kidney; 320 for the male brain), and the bottom 5% shortest proteins (*n* = 264 for the male kidney; 320 for the male brain). *P*-values were calculated using two-tailed Mann–Whitney *U* tests.

To investigate the physiological role of RPL41 *in vivo*, we generated Rpl41 KO (*Rpl41^−/−^*) mice in which the entire CDS of RPL41 was deleted using the CRISPR-Cas9 system (Supplementary Fig. S7A). When heterozygous mice were intercrossed, the number of homozygous *Rpl41^−/−^* pups at postnatal day 21 was lower than the expected Mendelian ratio (Fig. 7A). A chi-square goodness-of-fit test confirmed a marked deviation from the expected ratio (*χ*^2^ = 54.3, *P* = 1.6 × 10*^−^*^12^), attributable to a substantial reduction in *Rpl41^−/−^* offspring. Additionally, both male and female *Rpl41^−/−^* mice exhibited significantly reduced body weights compared with WT (*Rpl41^+/+^*) littermates (Fig. 7B and C). Consistent with this, tissue weight analysis revealed significant reductions in multiple organs and tissues, including the brain, kidney, spleen, epididymal fat pad, quadriceps femoris, and testis, although histological analysis revealed no overt morphological abnormalities (Supplementary Fig. S7B and C).

At embryonic day 17.5, the distribution of genotypes conformed to Mendelian expectations (*χ*^2^ = 0.47, *P* = .789), indicating that embryonic lethality was not evident at this stage (Supplementary Fig. S7D). However, placental weights were significantly reduced (Supplementary Fig. S7E and F). These findings suggest that developmental defects during fetal life, potentially linked to impaired placental function, may contribute to the reduced survival and growth retardation observed postnatally in *Rpl41^−/−^* mice.

### Altered translational dynamics and reduced expression of long proteins in Rpl41-deficient tissues

To assess the impact of RPL41 deficiency on translation *in vivo*, we first examined polysome profiles using kidney extracts, as this organ showed a marked reduction in size. In *Rpl41^−/−^* kidneys, polysome profiling revealed a significant increase in the 60S/40S ratio (Fig. 7D). Next, we performed Ribo-seq using lysates from the kidneys and brains of 10-week-old male mice. Biological replicates showed high reproducibility, and ribosomal footprints were predominantly mapped to CDSs with clear three-nucleotide periodicities, confirming the generation of high-quality libraries (Supplementary Fig. S8A and B; Supplementary Table S4). Metagene analysis revealed no major shifts in footprint distribution across the 5′-UTR, CDS, or 3′-UTR upon RPL41 loss (Supplementary Fig. S8C). However, unlike HEK293T cells, RPL41 deficiency resulted in significant changes in TE for a substantial number of transcripts in both the kidney and brain (Fig. 7E and F; Supplementary Table S14). In contrast, codon-level ribosome occupancy analysis showed only modest differences that were less pronounced than those observed in HEK293T cells (Supplementary Fig. S8D). Additional analyses of transcriptome-wide changes, including RNA-seq MA plots, comparisons of RNA-seq and Ribo-seq fold changes, and GO enrichment of TE-altered genes, are shown in Supplementary Fig. S9.

Consistent with the cell-based data, the size distribution of RPFs was altered in both kidney and brain tissues of *Rpl41^−/−^* mice (Fig. 7G and H). Quantification of the ratio of short to long footprints showed a significant reduction in kidney, whereas the difference in brain did not reach statistical significance (Fig. 7I). To evaluate ribosome collisions, we performed disome-seq using kidney lysates. We did not detect a statistically significant global difference between genotypes (Fig. 7J and Supplementary Table S15). These results indicate that, in mouse tissues as in HEK293T cells, loss of RPL41 alters ribosome state distributions but does not produce a robust global change in disome abundance under our experimental conditions.

Finally, to determine whether long protein homeostasis was similarly affected *in vivo*, we conducted DIA-MS analysis of kidney and brain lysates. Although the overall number of differentially expressed proteins was limited, with no significant changes observed in RPs (Fig. 7K and L; Supplementary Table S16), a comparison of the top 5% longest and bottom 5% shortest proteins revealed significantly reduced expression of the longest proteins and a relative increase in the shortest proteins (Fig. 7M and N). In addition, length-stratified RNA-seq analysis showed modest decreases in transcripts encoding long proteins in both kidney and brain (Supplementary Fig. S10A), suggesting that RNA-level changes may partly contribute to their reduced protein abundance. By contrast, RPF abundance and TE were significantly increased for transcripts encoding the longest proteins in both tissues (Supplementary Fig. S10B and C). Because increased RPF abundance can reflect increased ribosome occupancy or prolonged ribosome residence caused by slower elongation, rather than increased productive protein synthesis, these results do not indicate enhanced production of long proteins. Instead, the discordance between increased ribosome engagement and reduced steady-state protein abundance suggests that translation of long ORFs is less efficiently converted into stable protein output in *Rpl41^−/−^* tissues.

Taken together, these results highlight the conserved role of RPL41 in modulating translational dynamics and supporting long-protein homeostasis in mammalian tissues.

## Discussion

In this study, we demonstrated that the loss of RPL41 in cultured human cells and mice leads to multiple translational defects, including alterations in A-site dynamics, reduced elongation speed, increased amino acid misincorporation, and impairment of long-protein homeostasis characterized by reduced abundance and increased insolubility.

Consistent with a previous systematic knockdown screen showing that RPL41 depletion did not markedly affect cell cycle progression [64], complete RPL41 KO did not significantly impair cell proliferation under our experimental conditions. Structurally, RPL41 contributes to the eukaryote-specific bridge eB14 near the decoding center. Cryo-EM analysis showed that loss of RPL41 did not induce major static rearrangements of the averaged ribosome structure, but instead increased conformational heterogeneity of the 80S ribosome, particularly in the relative positioning of the 40S subunit. These findings suggest that RPL41 helps constrain intersubunit dynamics and thereby supports efficient translation.

Our results demonstrate that RPL41, a RP present predominantly in eukaryotes although retained in some archaea, contributes to long-protein homeostasis. This is particularly relevant given the evolutionary tendency of eukaryotic proteins to be structurally more complex than prokaryotic proteins. Comparative analyses across species have shown that although the average protein length is not drastically greater in eukaryotes, their proteomes contain a higher proportion of long proteins, often associated with multi-domain architectures and intrinsically disordered regions that extend length and expand functional diversity [65–67]. In this context, the conservation of RPL41 may help stabilize ribosomal dynamics and support the productive synthesis, folding, and solubility of structurally complex proteins. Thus, RPL41 may represent an adaptation that helps align ribosome function with the proteostatic demands of eukaryotic proteomes.

Polysome profiling revealed an altered balance of ribosomal species in both HEK293T cells and mouse kidneys, characterized by a relative increase in free 60S subunits. Cryo-EM analysis provided a structural framework for interpreting this phenotype. Rather than supporting a model of simple bulk dissociation of 80S ribosomes, the structural data indicate that RPL41-deficient ribosomes display increased conformational heterogeneity, with multiple 80S subclasses showing altered 40S positioning and, in some cases, weakened 40S density. Thus, the primary consequence of RPL41 loss appears to be destabilized relative positioning of the 40S subunit rather than complete separation of the two ribosomal subunits.

The relative accumulation of free 60S subunits may therefore reflect a shift in the equilibrium among ribosomal states in the absence of RPL41. Because this biochemical phenotype is not readily explained by widespread ribosome dissociation during elongation, it is possible that loss of RPL41 also influences additional processes, such as ribosome remodeling, surveillance, or state-specific turnover, that are not captured by the current structural snapshots. Although the present data do not define the underlying mechanism, they suggest that the consequences of RPL41 loss extend beyond a simple dissociation model. This interpretation is consistent with the ribosome profiling data, which showed no change in polarity scores, including when transcripts were stratified by encoded protein length, and therefore no evidence for widespread ribosome drop-off along CDSs. Transcripts encoding the longest proteins showed statistically significant but quantitatively modest reductions in TE and RPF abundance, which may contribute to, but do not fully explain, the more pronounced reduction in long-protein abundance. Pulse-SILAC analysis likewise did not support accelerated turnover of long proteins. In contrast, long proteins were significantly enriched in the insoluble fraction. Together, these results suggest that the selective reduction of long proteins cannot be attributed to a single uniform decrease in translational output or preferential degradation. Rather, modest defects in translational output, elongation kinetics, and fidelity may cumulatively compromise the productive synthesis, folding, and solubility of long proteins.

The reduced polysome/monosome ratio, slower elongation, and lack of a detectable reduction in bulk OP-puro incorporation reflect distinct aspects of translation and are not necessarily contradictory. Polysome abundance represents a steady-state balance influenced by translation initiation, elongation, termination, ribosome recycling, and ribosomal subunit availability. Accordingly, the reduced polysome/monosome ratio in RPL41-deficient cells cannot be attributed to slower elongation alone and may also reflect altered ribosome loading or subunit homeostasis. By contrast, the harringtonine run-off assay directly measures ribosome traversal along CDSs, whereas OP-puro incorporation provides a bulk readout of nascent-chain labeling across the cell population. The modest elongation defect and altered polysome organization observed here may therefore be buffered at the level of total nascent protein synthesis while still affecting proteins that are particularly sensitive to cumulative translational and folding defects.

Loss of RPL41 was associated with reduced translational elongation speed. Translation rates vary among mouse tissues, with reported values of 6.8, 5.0, and 4.3 amino acids per second in the liver, kidney, and skeletal muscle, respectively [68]. Moreover, elongation rates declined by ∼20% between young and middle-aged mice, indicating that the translational speed was finely tuned in a tissue- and age-dependent manner. These observations highlight the physiological importance of maintaining elongation rates within an appropriate range and raise the possibility that RPL41 contributes to this regulation. It is also plausible that cells harbor ribosome populations, both with and without RPL41, introducing an additional layer of ribosome heterogeneity that may help maintain proteostasis. However, detecting this heterogeneity remains challenging because RPL41 is an unusually small and highly basic peptide, which precludes its reliable detection using conventional MS. Emerging approaches, such as ribosome expansion microscopy, which enables single-ribosome visualization [8], may help reveal the distribution and dynamics of RPL41-containing ribosomes *in vivo*.

Ribosomal heterogeneity has also been documented in other contexts. For example, in striated muscle, RPL3L/uL3L is incorporated into ribosomes instead of RPL3, and RPL3L-containing ribosomes are required for the efficient translation of transcripts encoding proteins critical for cardiac contraction [12]. Additionally, partial loss of specific RP genes in mice can produce diverse developmental phenotypes. For instance, Rpl38/eL38 promotes selective translation of homeobox transcripts and is essential for proper body patterning [69], whereas Rplp1 haploinsufficiency causes male infertility and defects in brain development [70]. In humans, mutations in RP genes are linked to a range of congenital disorders: RPL21 mutations cause congenital hypotrichosis, RPSA mutations underlie isolated congenital asplenia [71, 72], and Diamond–Blackfan anemia is associated with mutations in multiple RP genes, including RPS19, RPS24/eS24, RPS17/eS17, and RPL35A/eL33 [73]. Taken together, these studies reinforce the emerging paradigm that individual RPs perform distinct, non-redundant functions beyond their canonical roles in bulk translation.

In this study, we defined the mammalian cellular functions of RPL41 and established its importance in maintaining ribosome dynamics, translational fidelity, and long-protein homeostasis. Further elucidation of whether RPL41-deficient ribosomes are actively employed as part of ribosomal heterogeneity will provide deeper insights into translational regulation and expand our understanding of fundamental cell biology.

## Supplementary Material

gkag797_Supplemental_Files

## Data Availability

All sequence data were deposited in Gene Expression Omnibus under accession number GSE308359. All raw MS data were stored in the jPOSTrepo (https://repository.jpostdb.org/). The jPOSTIDs/PXIDs for projects containing these data were JPST004073/PXD068398, JPST004076/PXD068399, and JPST004574/PXD079032. Cryo-EM maps and the corresponding atomic models have been deposited in the Electron Microscopy Data Bank (EMDB) and Protein Data Bank (PDB) with the following accession codes: non-rotated 80S (EMD-66297, PDB 9WW4), rotated 80S (EMD-66298, PDB 9WW5), non-rotated ΔRPL41 80S (EMD-66299, PDB 9WW6), and rotated ΔRPL41 80S (EMD-66301, PDB 9WW7). All custom Python scripts used for the analyses in this study are available upon request.

## References

[1] Thomson E, Ferreira-Cerca S, Hurt E. Eukaryotic ribosome biogenesis at a glance. J Cell Sci. 2013;126:4815–21. 10.1242/jcs.111948. 24172536

[2] Shi Z, Fujii K, Kovary KM et al. Heterogeneous ribosomes preferentially translate distinct subpools of mRNAs genome-wide. Mol Cell. 2017;67:71–83.e7. 10.1016/j.molcel.2017.05.021. 28625553 PMC5548184

[3] Norris K, Hopes T, Aspden JL. Ribosome heterogeneity and specialization in development. WIREs RNA. 2021;12:e1644. 10.1002/wrna.1644. 33565275 PMC8647923

[4] Genuth NR, Barna M. The discovery of ribosome heterogeneity and its implications for gene regulation and organismal life. Mol Cell. 2018;71:364–74. 10.1016/j.molcel.2018.07.018. 30075139 PMC6092941

[5] Komili S, Farny NG, Roth FP et al. Functional specificity among ribosomal proteins regulates gene expression. Cell. 2007;131:557–71. 10.1016/j.cell.2007.08.037. 17981122 PMC2443060

[6] Gay DM, Lund AH, Jansson MD. Translational control through ribosome heterogeneity and functional specialization. Trends Biochem Sci. 2022;47:66–81. 10.1016/j.tibs.2021.07.001. 34312084

[7] Li D, Wang J. Ribosome heterogeneity in stem cells and development. J Cell Biol. 2020;219:e202001108. 10.1083/jcb.202001108. 32330234 PMC7265316

[8] Zhang Z, Xu A, Bai Y et al. A subcellular map of translational machinery composition and regulation at the single-molecule level. Science. 2025;387:eadn2623. 40048539 10.1126/science.adn2623 PMC13102253

[9] Folgado-Marco V, Ames K, Chuen J et al. Haploinsufficiency of the essential gene Rps12 causes defects in erythropoiesis and hematopoietic stem cell maintenance. eLife. 2023;12:e69322. 10.7554/eLife.69322. 37272618 PMC10287158

[10] Matsson H, Davey EJ, Draptchinskaia N et al. Targeted disruption of the ribosomal protein S19 gene is lethal prior to implantation. Mol Cell Biol. 2004;24:4032–7. 10.1128/MCB.24.9.4032-4037.2004. 15082795 PMC387766

[11] Genuth NR, Shi Z, Kunimoto K et al. A stem cell roadmap of ribosome heterogeneity reveals a function for RPL10A in mesoderm production. Nat Commun. 2022;13:1–19. 10.1038/s41467-022-33263-3. 36123354 PMC9485161

[12] Shiraishi C, Matsumoto A, Ichihara K et al. RPL3L-containing ribosomes determine translation elongation dynamics required for cardiac function. Nat Commun. 2023;14:1–17. 10.1038/s41467-023-37838-6. 37080962 PMC10119107

[13] Aravindan RG, Kirn-Safran CB, Smith MA et al. Ultrastructural changes and asthenozoospermia in murine spermatozoa lacking the ribosomal protein L29/HIP gene. Asian J Androl. 2014;16:925. 25155104 10.4103/1008-682X.133318 PMC4236347

[14] Sloofman LG, Verdelis K, Spevak L et al. Effect of HIP/ribosomal protein L29 deficiency on mineral properties of murine bones and teeth. Bone. 2010;47:93–101. 10.1016/j.bone.2010.03.015. 20362701 PMC2892198

[15] Kirn-Safran CB, Oristian DS, Focht RJ et al. Global growth deficiencies in mice lacking the ribosomal protein HIP/RPL29. Dev Dyn. 2007;236:447–60. 10.1002/dvdy.21046. 17195189

[16] Oristian DS, Slooíman LG, Zhou X et al. Ribosomal protein L29/HIP deficiency delays osteogenesis and increases fragility of adult bone in mice. J Orthop Res. 2009;27:28–35. 10.1002/jor.20706. 18661500 PMC2644558

[17] Fahl SP, Sertori R, Zhang Y et al. Loss of ribosomal protein paralog Rpl22-Like1 blocks lymphoid development without affecting protein synthesis. J Immunol. 2022;208:870–80. 10.4049/jimmunol.2100668. 35046107 PMC8827804

[18] Zhang Y, O’Leary MN, Peri S et al. Ribosomal proteins Rpl22 and Rpl22l1 control morphogenesis by regulating pre-mRNA splicing. Cell Rep. 2017;18:545–56. 10.1016/j.celrep.2016.12.034. 28076796 PMC5234864

[19] O’Leary MN, Schreiber KH, Zhang Y et al. The ribosomal protein Rpl22 controls ribosome composition by directly repressing expression of its own paralog. PLoS Genet. 2013;9:e1003708. 23990801 10.1371/journal.pgen.1003708 PMC3750023

[20] Anderson SJ, Lauritsen JPH, Hartman MG et al. Ablation of ribosomal protein L22 selectively impairs αβ T cell development by activation of a p53-dependent checkpoint. Immunity. 2007;26:759–72. 10.1016/j.immuni.2007.04.012. 17555992

[21] Lecompte O, Ripp R, Thierry JC et al. Comparative analysis of ribosomal proteins in complete genomes: an example of reductive evolution at the domain scale. Nucleic Acids Res. 2002;30:5382–90. 10.1093/nar/gkf693. 12490706 PMC140077

[22] Ben-Shem A, De Loubresse NG, Melnikov S et al. The structure of the eukaryotic ribosome at 3.0 Å resolution. Science. 2011;334:1524–9. 10.1126/science.1212642. 22096102

[23] Khatter H, Myasnikov AG, Natchiar SK et al. Structure of the human 80S ribosome. Nature. 2015;520:640–5. 10.1038/nature14427. 25901680

[24] Yu X, Warner JR. Expression of a micro-protein. J Biol Chem. 2001;276:33821–5. 10.1074/jbc.M103772200. 11451953

[25] Tamm T, Kisly I, Remme J. Functional interactions of ribosomal intersubunit bridges in *Saccharomyces cerevisiae*. Genetics. 2019;213:1329–39. 10.1534/genetics.119.302777. 31649153 PMC6893367

[26] Dresios J, Panopoulos P, Suzuki K et al. A dispensable yeast ribosomal protein optimizes peptidyltransferase activity and affects translocation. J Biol Chem. 2003;278:3314–22. 10.1074/jbc.M207533200. 12433929

[27] Meskauskas A, Harger JW, Jacobs KLM et al. Decreased peptidyltransferase activity correlates with increased programmed -1 ribosomal frameshifting and viral maintenance defects in the yeast *Saccharomyces cerevisiae*. RNA. 2003;9:982–92. 10.1261/rna.2165803. 12869709 PMC1240118

[28] Ran FA, Hsu PD, Wright J et al. Genome engineering using the CRISPR-Cas9 system. Nat Protoc. 2013;8:2281–308. 10.1038/nprot.2013.143. 24157548 PMC3969860

[29] Miyoshi H, Blömer U, Takahashi M et al. Development of a self-inactivating lentivirus vector. J Virol. 1998;72:8150–7. 10.1128/JVI.72.10.8150-8157.1998. 9733856 PMC110156

[30] Juszkiewicz S, Hegde RS. Initiation of quality control during poly(A) translation requires site-specific ribosome ubiquitination. Mol Cell. 2017;65:743–50.e4. 10.1016/j.molcel.2016.11.039. 28065601 PMC5316413

[31] Muldoon-Jacobs KL, Dinman JD. Specific effects of ribosome-tethered molecular chaperones on programmed -1 ribosomal frameshifting. Euk Cell. 2006;5:762–70. 10.1128/EC.5.4.762-770.2006. PMC1459665 16607023

[32] Mastronarde DN . Automated electron microscope tomography using robust prediction of specimen movements. J Struct Biol. 2005;152:36–51. 10.1016/j.jsb.2005.07.007. 16182563

[33] Punjani A, Rubinstein JL, Fleet DJ et al. cryoSPARC: algorithms for rapid unsupervised cryo-EM structure determination. Nat Methods. 2017;14:290–6. 10.1038/nmeth.4169. 28165473

[34] Punjani A, Zhang H, Fleet DJ. Non-uniform refinement: adaptive regularization improves single-particle cryo-EM reconstruction. Nat Methods. 2020;17:1214–21. 10.1038/s41592-020-00990-8. 33257830

[35] Pettersen EF, Goddard TD, Huang CC et al. UCSF Chimera—a visualization system for exploratory research and analysis. J Comput Chem. 2004;25:1605–12. 10.1002/jcc.20084. 15264254

[36] Emsley P, Lohkamp B, Scott WG et al. Features and development of Coot. Acta Crystallogr D Biol Crystallogr. 2010;66:486–501. 10.1107/S0907444910007493. 20383002 PMC2852313

[37] Holvec S, Barchet C, Lechner A et al. The structure of the human 80S ribosome at 1.9 Å resolution reveals the molecular role of chemical modifications and ions in RNA. Nat Struct Mol Biol. 2024;31:1251–64. 10.1038/s41594-024-01274-x. 38844527

[38] Adams PD, Afonine PV, Bunkóczi G et al. PHENIX: a comprehensive Python-based system for macromolecular structure solution. Acta Crystallogr D Biol Crystallogr. 2010;66:213–21. 10.1107/S0907444909052925. 20124702 PMC2815670

[39] Goddard TD, Huang CC, Meng EC et al. UCSF ChimeraX: meeting modern challenges in visualization and analysis. Protein Sci. 2018;27:14–25. 10.1002/pro.3235. 28710774 PMC5734306

[40] O’Connor PBF, Andreev DE, Baranov PV. Comparative survey of the relative impact of mRNA features on local ribosome profiling read density. Nat Commun. 2016;7:12915. 27698342 10.1038/ncomms12915 PMC5059445

[41] Neumann T, Herzog VA, Muhar M et al. Quantification of experimentally induced nucleotide conversions in high-throughput sequencing datasets. BMC Bioinformatics. 2019;20:1–16. 10.1186/s12859-019-2849-7. 31109287 PMC6528199

[42] Kawashima Y, Nagai H, Konno R et al. Single-shot 10K proteome approach: over 10,000 protein identifications by data-independent acquisition-based single-shot proteomics with ion mobility spectrometry. J Proteome Res. 2022;21:1418–27. 10.1021/acs.jproteome.2c00023. 35522919 PMC9171847

[43] Schwanhüusser B, Busse D, Li N et al. Global quantification of mammalian gene expression control. Nature. 2011;473:337–42. 10.1038/nature10098. 21593866

[44] Zhong Y, Karaletsos T, Drewe P et al. RiboDiff: detecting changes of mRNA translation efficiency from ribosome footprints. Bioinformatics. 2017;33:139–41. 10.1093/bioinformatics/btw585. 27634950 PMC5198522

[45] Ingolia NT, Ghaemmaghami S, Newman JRS et al. Genome-wide analysis *in vivo* of translation with nucleotide resolution using ribosome profiling. Science. 2009;324:218–23. 10.1126/science.1168978. 19213877 PMC2746483

[46] McGlincy NJ, Ingolia NT. Transcriptome-wide measurement of translation by ribosome profiling. Methods. 2017;126:112–29. 10.1016/j.ymeth.2017.05.028. 28579404 PMC5582988

[47] Ji Z . RibORF: identifying genome-wide translated open reading frames using ribosome profiling. Curr Protoc Mol Biol. 2018;124:e67. 30178897 10.1002/cpmb.67 PMC6168376

[48] Schuller AP, Wu CCC, Dever TE et al. eIF5A functions globally in translation elongation and termination. Mol Cell. 2017;66:194–205.e5. 10.1016/j.molcel.2017.03.003. 28392174 PMC5414311

[49] Wu CCC, Zinshteyn B, Wehner KA et al. High-resolution ribosome profiling defines discrete ribosome elongation states and translational regulation during cellular stress. Mol Cell. 2019;73:959–70.e5. 10.1016/j.molcel.2018.12.009e5. 30686592 PMC6411040

[50] Lareau LF, Hite DH, Hogan GJ et al. Distinct stages of the translation elongation cycle revealed by sequencing ribosome-protected mRNA fragments. eLife. 2014;3:e01257. 10.7554/eLife.01257. 24842990 PMC4052883

[51] Ingolia NT, Lareau LF, Weissman JS. Ribosome profiling of mouse embryonic stem cells reveals the complexity and dynamics of mammalian proteomes. Cell. 2011;147:789–802. 10.1016/j.cell.2011.10.002. 22056041 PMC3225288

[52] Joazeiro CAP . Mechanisms and functions of ribosome-associated protein quality control. Nat Rev Mol Cell Biol. 2019;20:368–83. 10.1038/s41580-019-0118-2. 30940912 PMC7138134

[53] Ikeuchi K, Izawa T, Inada T. Recent progress on the molecular mechanism of quality controls induced by ribosome stalling. Front Genet. 2019;9:743. 10.3389/fgene.2018.00743. 30705686 PMC6344382

[54] Juszkiewicz S, Speldewinde SH, Wan L et al. The ASC-1 complex disassembles collided ribosomes. Mol Cell. 2020;79:603–14.e8. 10.1016/j.molcel.2020.06.006. 32579943 PMC7447978

[55] Narita M, Denk T, Matsuo Y et al. A distinct mammalian disome collision interface harbors K63-linked polyubiquitination of uS10 to trigger hRQT-mediated subunit dissociation. Nat Commun. 2022;13:1–16. 10.1038/s41467-022-34097-9. 36302773 PMC9613687

[56] Best K, Ikeuchi K, Kater L et al. Structural basis for clearing of ribosome collisions by the RQT complex. Nat Commun. 2023;14:1–12. 10.1038/s41467-023-36230-8. 36801861 PMC9938168

[57] Hashimoto S, Sugiyama T, Yamazaki R et al. Identification of a novel trigger complex that facilitates ribosome-associated quality control in mammalian cells. Sci Rep. 2020;10:1–12. 10.1038/s41598-020-60241-w. 32099016 PMC7042231

[58] Meydan S, Guydosh NR. Disome and trisome profiling reveal genome-wide targets of ribosome quality control. Mol Cell. 2020;79:588–602.e6. 10.1016/j.molcel.2020.06.010. 32615089 PMC7484464

[59] Han P, Shichino Y, Schneider-Poetsch T et al. Genome-wide survey of ribosome collision. Cell Rep. 2020;31:107610. 10.1016/j.celrep.2020.107610. 32375038 PMC7746506

[60] Arpat AB, Liechti A, De Matos M et al. Transcriptome-wide sites of collided ribosomes reveal principles of translational pausing. Genome Res. 2020;30:985–99. 10.1101/gr.257741.119. 32703885 PMC7397865

[61] Kramer EB, Vallabhaneni H, Mayer LM et al. A comprehensive analysis of translational missense errors in the yeast Saccharomyces cerevisiae. RNA. 2010;16:1797–808. 10.1261/rna.2201210. 20651030 PMC2924539

[62] Nagai R, Milam OL, Niwa T et al. Ribosomal expansion segment contributes to translation fidelity via N-terminal processing of ribosomal proteins. Nucleic Acids Res. 2025;53:448. 10.1093/nar/gkaf448. PMC12117404 40433980

[63] Fujii K, Susanto TT, Saurabh S et al. Decoding the function of expansion segments in ribosomes. Mol Cell. 2018;72:1013–20.e6. 10.1016/j.molcel.2018.11.023. 30576652 PMC6407129

[64] Luan Y, Tang N, Yang J et al. Deficiency of ribosomal proteins reshapes the transcriptional and translational landscape in human cells. Nucleic Acids Res. 2022;50:6601–17. 10.1093/nar/gkac053. 35137207 PMC9262593

[65] Nevers Y, Glover NM, Dessimoz C et al. Protein length distribution is remarkably uniform across the tree of life. Genome Biol. 2023;24:1–20. 10.1186/s13059-023-02973-2. 37291671 PMC10251718

[66] Basile W, Salvatore M, Bassot C et al. Why do eukaryotic proteins contain more intrinsically disordered regions?. PLoS Comput Biol. 2019;15:e1007186. 10.1371/journal.pcbi.1007186. 31329574 PMC6675126

[67] Björklund ÅK, Ekman D, Elofsson A. Expansion of protein domain repeats. PLoS Comput Biol. 2006;2:e114. 10.1371/journal.pcbi.0020114. 16933986 PMC1553488

[68] Gerashchenko MV, Peterfi Z, Yim SH et al. Translation elongation rate varies among organs and decreases with age. Nucleic Acids Res. 2021;49:e9. 10.1093/nar/gkaa1103. 33264395 PMC7826258

[69] Kondrashov N, Pusic A, Stumpf CR et al. Ribosome-mediated specificity in Hox mRNA translation and vertebrate tissue patterning. Cell. 2011;145:383–97. 10.1016/j.cell.2011.03.028. 21529712 PMC4445650

[70] Perucho L, Artero-Castro A, Guerrero S et al. RPLP1, a crucial ribosomal protein for embryonic development of the nervous system. PLoS One. 2014;9:e99956. 10.1371/journal.pone.0099956. 24959908 PMC4069005

[71] Zhou C, Zang D, Jin Y et al. Mutation in ribosomal protein L21 underlies hereditary hypotrichosis simplex. Hum Mutat. 2011;32:710–4. 10.1002/humu.21503. 21412954

[72] Bolze A, Mahlaoui N, Byun M et al. Ribosomal protein SA haploinsufficiency in humans with isolated congenital asplenia. Science. 2013;340:976–8. 10.1126/science.1234864. 23579497 PMC3677541

[73] Boria I, Garelli E, Gazda HT et al. The ribosomal basis of diamond-blackfan anemia: mutation and database update. Hum Mutat. 2010;31:1269–79. 10.1002/humu.21383. 20960466 PMC4485435

