# Supplementary material for: RPL41 stabilizes ribosome dynamics and supports long-protein homeostasis in mammals
Source: Nucleic Acids Res. 2026 Aug 19;54(15):gkag797. doi: 10.1093/nar/gkag797 (PMC13485352; doi:10.1093/nar/gkag797)
Supplement: Supplementary file 1 [file gkag797_supplemental_files.zip › RPL41_10_suppl.pdf]

Supplementary Information

RPL41 stabilizes ribosome dynamics and supports long-protein homeostasis in mammals

Mina Hirata, Maho Fujino, Kazuya Ichihara, Ronghao Tang, Momoko Narita, Wakana Iwasaki, Chisa Shiraishi, Taichi Shiraishi, Atsushi Hatano, Toru Suzuki, Tokichiro Abe, Tomoyo Takami, Emi Mishiro-Sato, Atsushi Toyoda, Masaki Matsumoto, Yoshikazu Tanaka, Takuhiro Ito, Toshifumi Inada, Keiichi I. Nakayama, Takeshi Yokoyama, and Akinobu Matsumoto

Supplementary Table information

Supplementary Figures

Supplementary Table information (provided in separate Excel sheets)

Supplementary Table S1. Oligonucleotide sequences used in this study, related to Methods.

Supplementary Table S2. Cryo-EM data collection, image processing, model building and validation statistics.

Supplementary Table S3. Protein abundance in the soluble fraction of WT and RPL41 KO HEK293T cells measured by quantitative proteomics (DIA-MS), related to Fig. 1.

Supplementary Table S4. Three-nucleotide periodicity values from Ribo-seq in WT and RPL41 KO HEK293T cells, and in kidney and brain of *Rpl41*$^{+/+}$ and *Rpl41*$^{-/-}$ mice, related to Figs. 3 and 7.

Supplementary Table S5. Polarity scores for WT and RPL41 KO HEK293T cells, related to Fig. 3.

Supplementary Table S6. Translation efficiency (TE) values for WT and RPL41 KO HEK293T cells, related to Fig. 3.

Supplementary Table S7. Fold changes in ribosome-protected fragment (RPF) abundance and translation efficiency (TE) values for RQC-related genes in RPL41 KO HEK293T cells compared with WT cells, related to Fig. 4.

Supplementary Table S8. Disome intensity normalized by monosome intensity in WT and RPL41 KO HEK293T cells, related to Fig. 4.

Supplementary Table S9. Fold changes in mRNA abundance in RPL41 KO HEK293T cells compared with WT cells, related to Fig. 6.

Supplementary Table S10. Fold changes in RPF abundance in RPL41 KO HEK293T cells compared with WT cells, related to Fig. 6.

Supplementary Table S11. Half-lives of coding transcripts estimated by SLAM-seq analysis in WT and RPL41 KO HEK293T cells, related to Fig. 6.

Supplementary Table S12. Protein half-lives estimated by pulse-SILAC analysis in WT and RPL41 KO HEK293T cells, related to Fig. 6.

Supplementary Table S13. Protein abundance in the insoluble fraction of WT and RPL41 KO HEK293T cells measured by DIA-MS, related to Fig. 6.

Supplementary Table S14. Fold changes in mRNA abundance, RPF abundance, and TE in kidney and brain from *Rpl41*$^{-/-}$ mice compared with *Rpl41*$^{+/+}$ mice, related to Fig. 7 and Supplementary Fig. S10.

Supplementary Table S15. Disome intensity normalized by monosome intensity in kidney from *Rpl41*$^{+/+}$ and *Rpl41*$^{-/-}$ mice, related to Fig. 7.

Supplementary Table S16. Protein abundance in the soluble fraction of kidney and brain from *Rpl41*$^{+/+}$ and *Rpl41*$^{-/-}$ mice measured by DIA-MS, related to Fig. 7.

Supplementary Figures

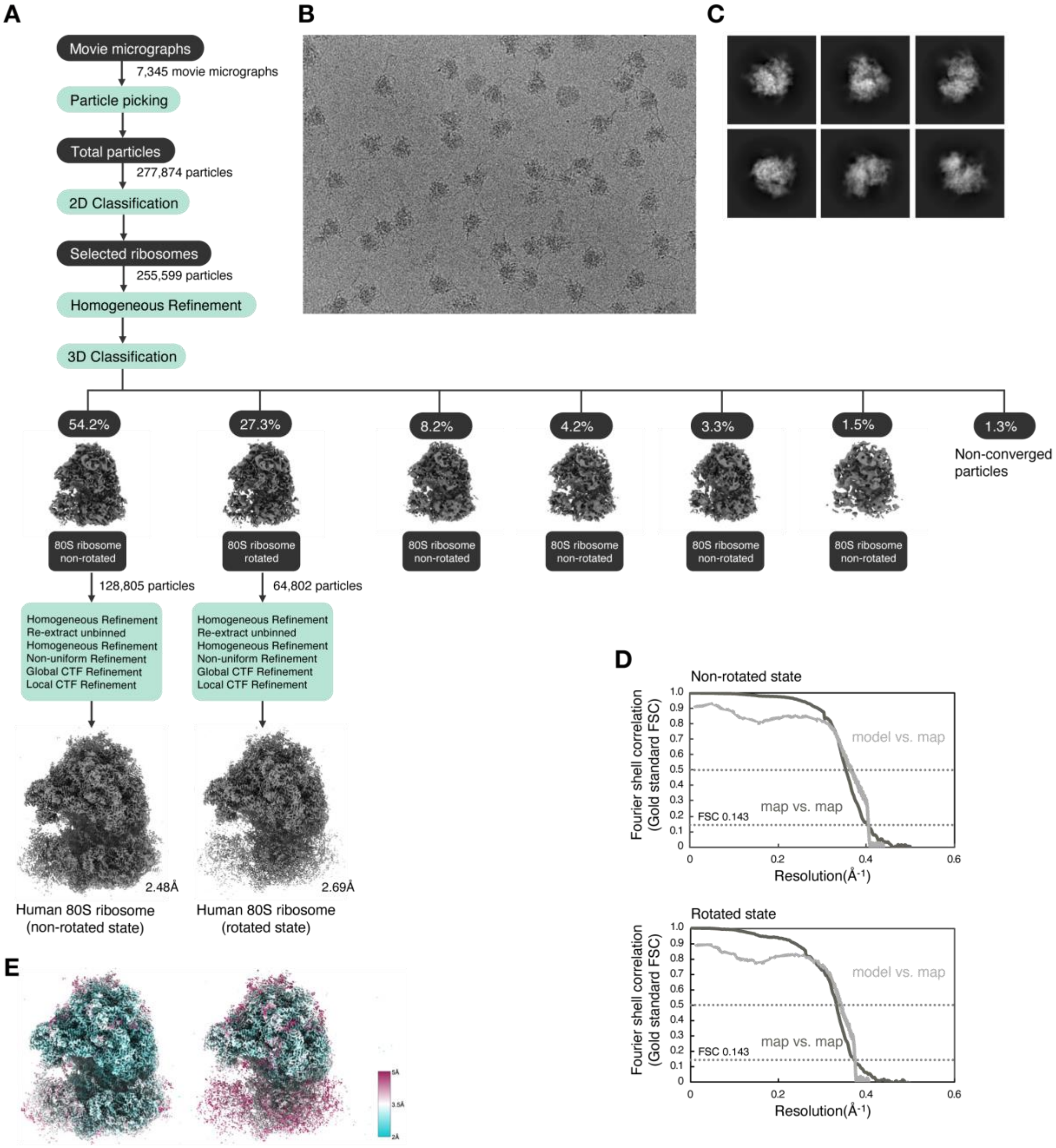

Hirata *et al.* Supplementary Figure 1

Supplementary Figure S1 Cryo-EM image processing workflow of the human wild-type 80S ribosome.

(**A**) Workflow of single-particle analysis of the human wild-type 80S ribosome using CryoSPARC. From 7,345 patch motion–corrected micrographs, 277,874 ribosome particles were picked. After 2D classification, 255,599 well-converged particles were retained and subjected to homogeneous refinement to obtain a consensus reconstruction. Subsequent 3D classification into 7 classes revealed two major populations corresponding to the

non-rotated (54.2%) and rotated (27.3%) 80S states. These classes were further refined by homogeneous, non-uniform, and CTF refinement, yielding cryo-EM maps at 2.48 Å (non-rotated) and 2.69 Å (rotated).

(**B**) Representative cryo-EM micrograph.

(**C**) Representative 2D class averages.

(**D**) Resolution curves: gold-standard Fourier shell correlation (FSC) between half-maps (brown) and FSC between the map and model (grey).

(**E**) Local resolution distribution of the cryo-EM reconstructions.

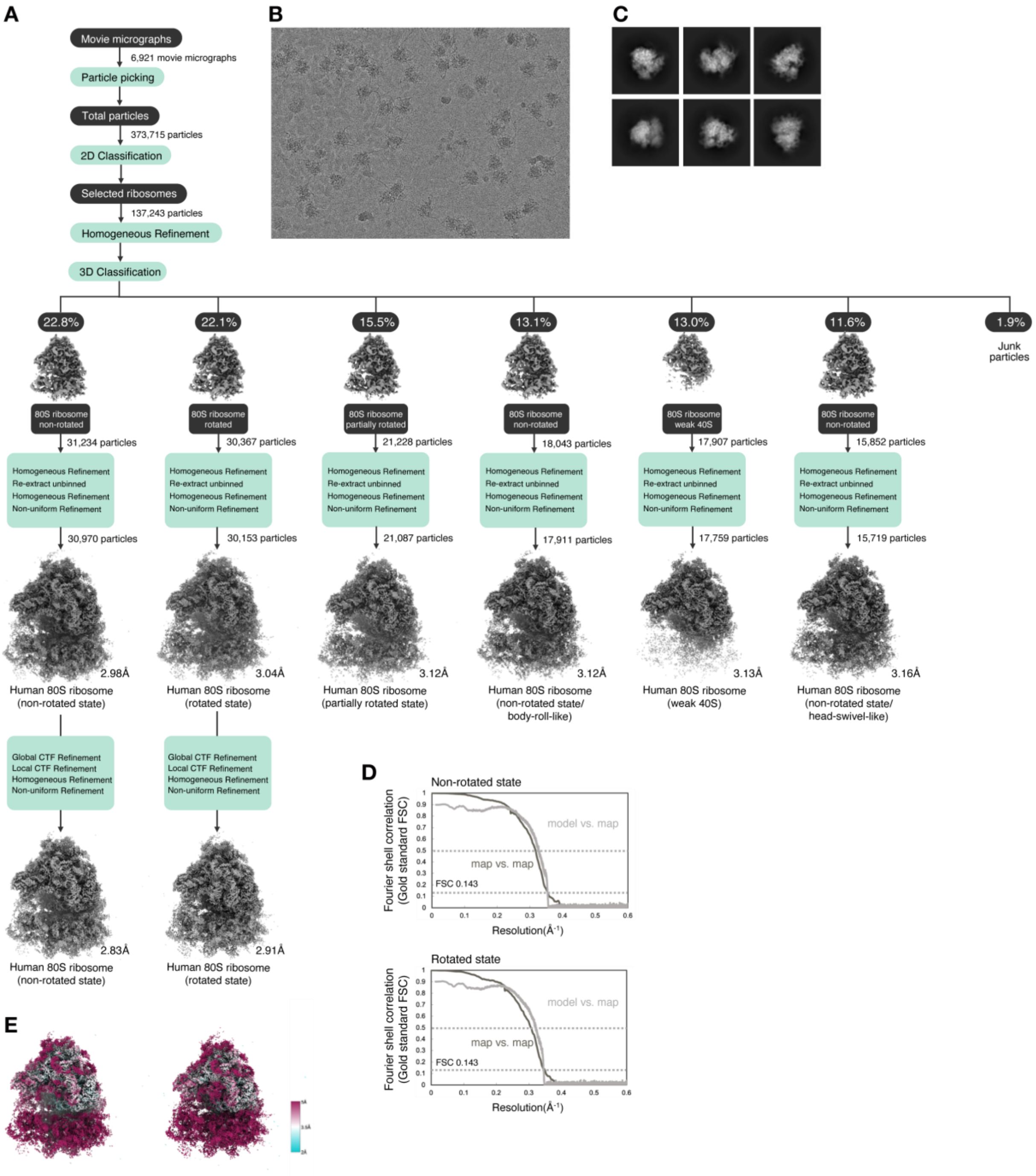

Hirata *et al.* Supplementary Figure 2

Supplementary Figure S2 Cryo-EM image processing workflow of the human RPL41-deleted 80S ribosome.

(**A**) Workflow of single-particle analysis of the RPL41-deleted mutant ribosome using CryoSPARC. From 6,921 patch motion–corrected micrographs, 373,715 particles were picked. After 2D classification, 137,243 well-converged ribosomal particles were retained, and subjected to homogeneous refinement to obtain a consensus reconstruction. Subsequent 3D classification into 7 classes identified six converged major populations: non-rotated 80S (22.8%), rotated 80S (22.1%), partially-rotated 80S (15.5%), non-rotated

80S with body roll (13.1%), 80S with weak 40S (13.0%), and non-rotated 80S with head swivel (11.6%). Six classes were further refined by homogeneous, non-uniform refinement. Subsequently, two major classes were further refined yielding cryo-EM maps at 2.83 Å (non-rotated), 2.91 Å (rotated).

(**B**) Representative cryo-EM micrograph.

(**C**) Representative 2D class averages.

(**D**) Resolution curves: gold-standard Fourier shell correlation (FSC) between half-maps (brown) and FSC between the map and model (grey).

(**E**) Local resolution distribution of the cryo-EM reconstructions.

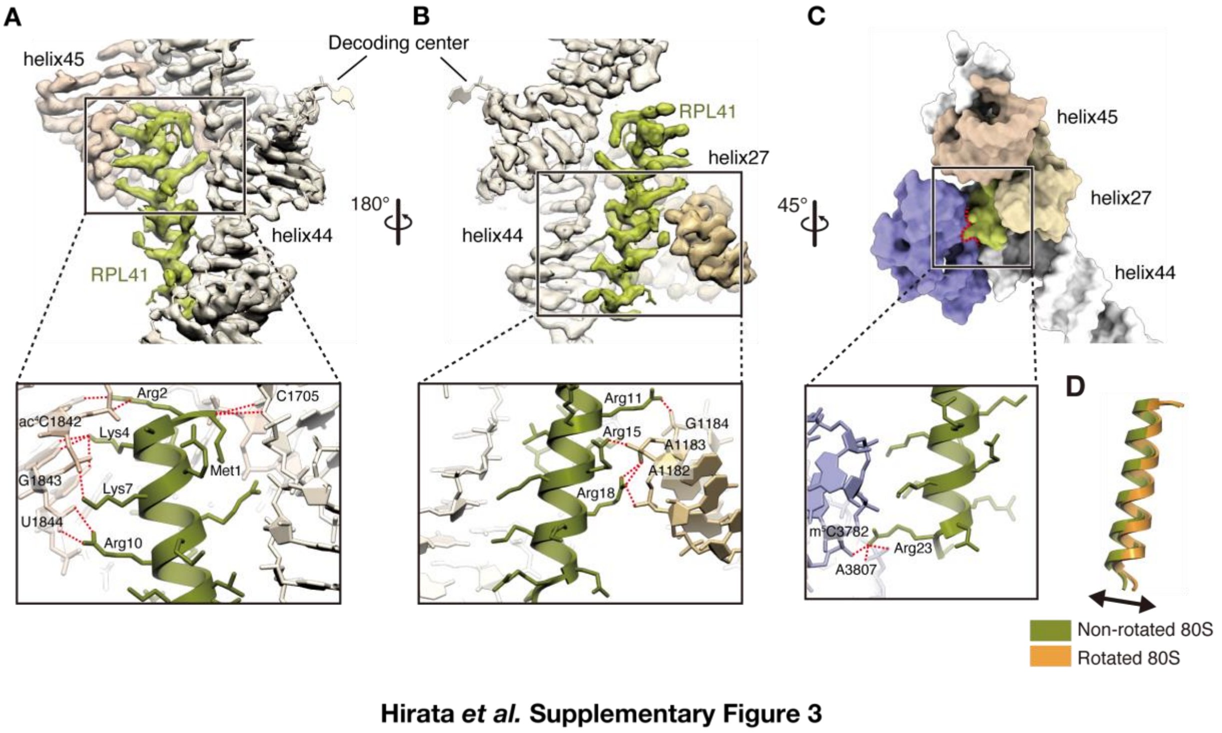

Supplementary Figure S3 Structural positioning of RPL41 at the eukaryote-specific intersubunit bridge eB14.

(**A** and **B**) Close-up views of RPL41 in the WT ribosome. The N-terminal region forms hydrogen bonds with helix 45 (A), while additional interactions with helix 27 stabilize its binding (B).

(**C**) Bridge formation between the 40S and 60S subunits. Arg23 at the C-terminus of RPL41 interacts with A3807 and m^5C3782 of the 28S rRNA, establishing the eB14 bridge.

(**D**) Comparison of RPL41 in rotated and non-rotated ribosomes. The C-terminus of RPL41 undergoes a slight positional shift during subunit rotation.

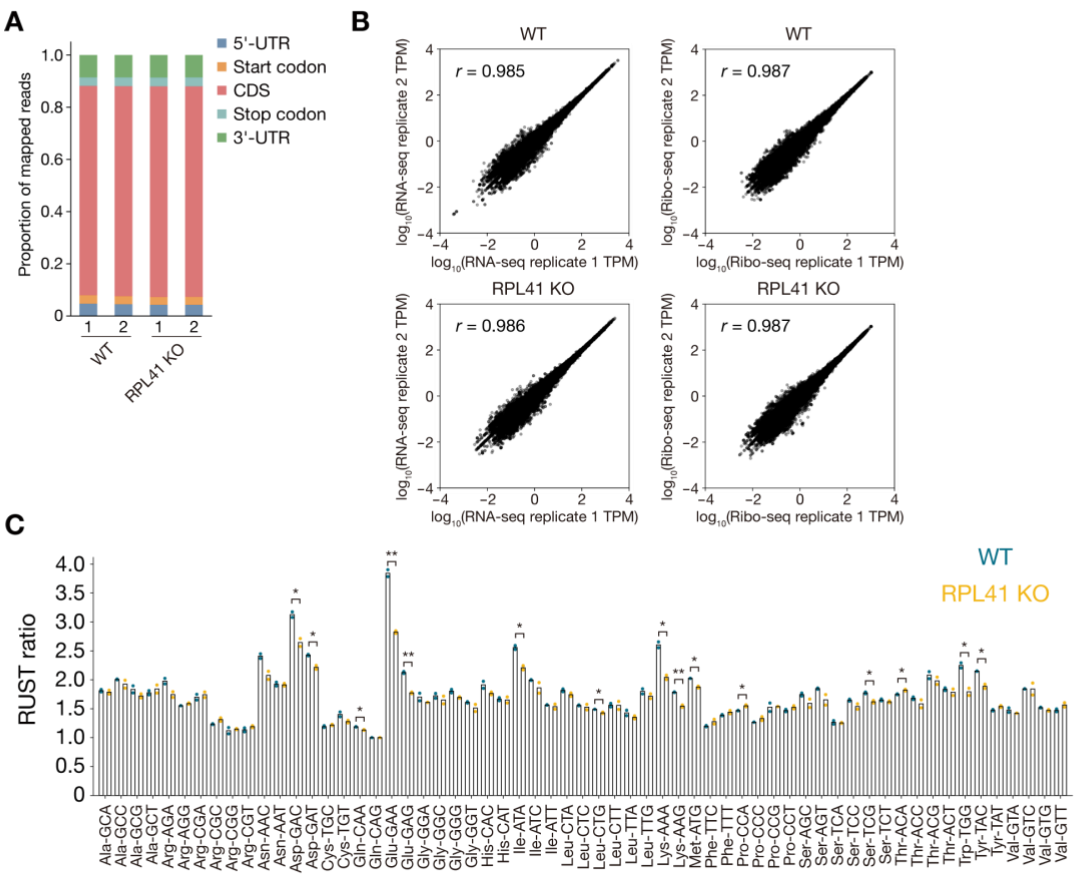

Hirata *et al.* Supplementary Figure 4

Supplementary Figure S4 Ribo-seq quality metrics and codon-level occupancy in RPL41-deficient cells.

(**A**) Distribution of ribosome-protected fragments (RPFs) across the 5′-UTR, start codon, coding sequence (CDS), stop codon, and 3′-UTR in HEK293T wild-type (WT) and RPL41 KO cells ($n = 2$).

(**B**) Correlation of TPM values from RNA-seq and Ribo-seq between biological replicates. Pearson's correlation coefficient (r) is indicated.

(**C**) RUST ratios at the level of individual codons in WT and RPL41 KO cells ($n = 2$). *$P < 0.05$, **$P < 0.01$ (two-tailed Student's t test).

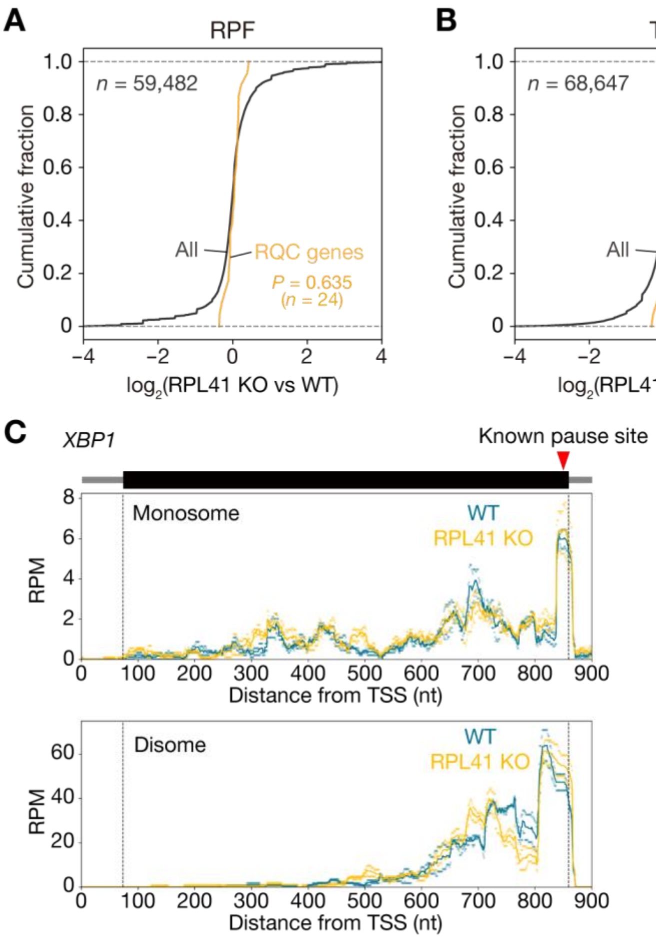

Hirata *et al.* Supplementary Figure 5

Supplementary Figure S5 RQC-related RPF abundance and translation efficiency and endogenous XBP1 ribosome collision profiles.

(**A** and **B**) Cumulative distributions of fold changes in ribosome-protected fragment (RPF) abundance (A) and translation efficiency (TE) (B) in RPL41 KO versus WT cells ($n = 2$). Data are shown for all transcripts and for transcripts encoding 24 RQC-related genes. *P*-values were calculated using a two-tailed Mann–Whitney *U* test.

(**C**) Read aggregation plots of monosome (Ribo-seq) and disome (disome-seq) footprints across the XBP1 transcript in WT and RPL41 KO cells. The known pause site is indicated.

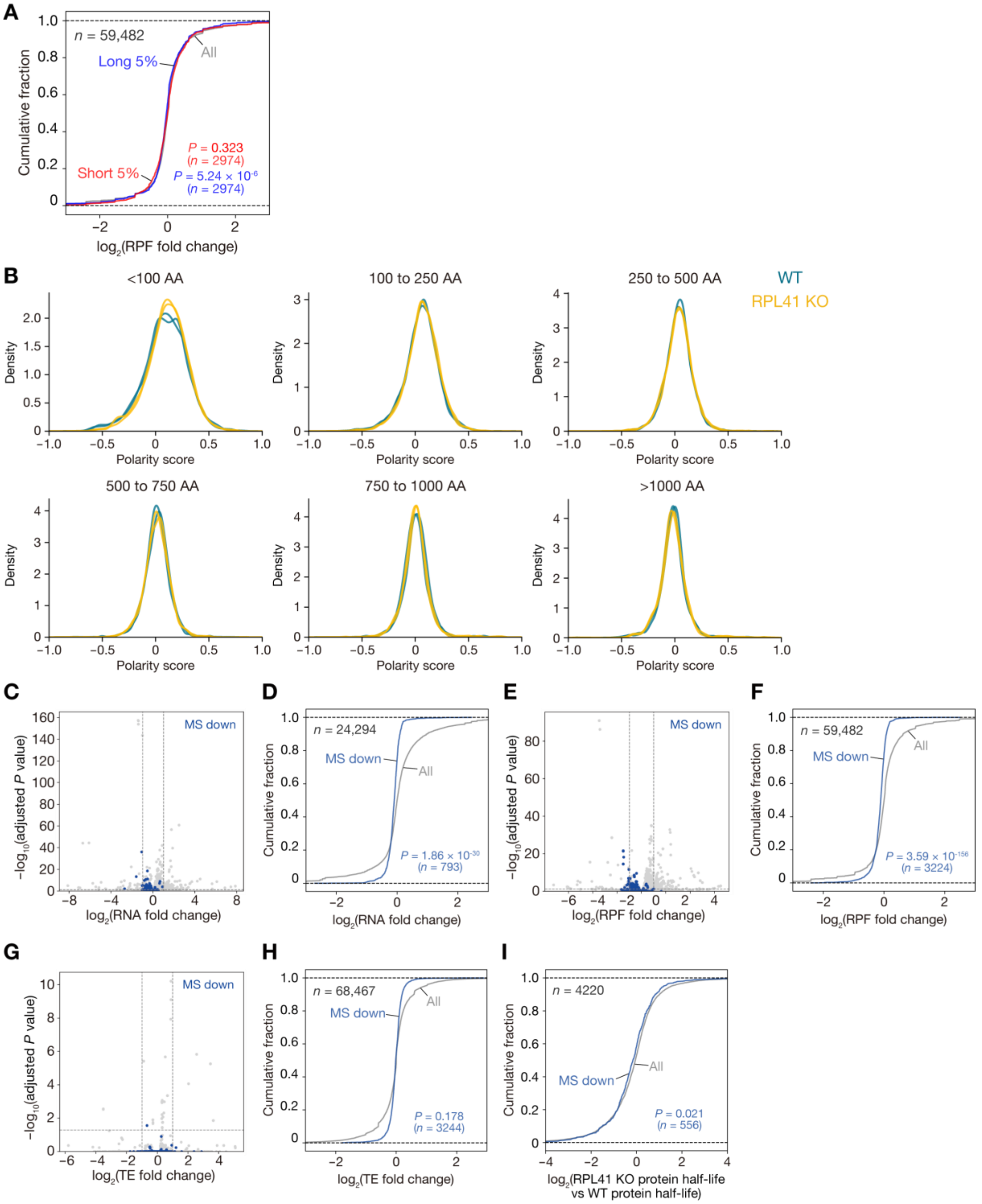

Hirata *et al.* Supplementary Figure 6

Supplementary Figure S6 Length-stratified RPF and polarity analyses and integrative analysis of proteins reduced in RPL41-deficient cells.

(**A**) Cumulative distributions of fold changes in RPF abundance between RPL41 KO and WT cells (n = 2). Shown are all detected transcripts (n = 59,482), transcripts encoding the

top 5% longest proteins (*n* = 2,974), and transcripts encoding the bottom 5% shortest proteins (*n* = 2,974). *P*-values were calculated using a two-tailed Mann–Whitney *U* test.
(**B**) Distributions of polarity scores for transcripts grouped according to encoded protein length in WT and RPL41 KO cells.
(**C–H**) Integrative analysis of proteins whose abundance was decreased in RPL41 KO cells by DIA-MS. Volcano plots and cumulative distributions are shown for RNA abundance (C and D), RPF abundance (E and F), and TE (G and H). In the volcano plots, transcripts corresponding to MS-down proteins are highlighted in blue. In the cumulative distributions, data are shown for all detected transcripts and for transcripts corresponding to MS-down proteins. *P*-values were calculated using two-tailed Mann–Whitney *U* tests.
(**I**) Cumulative distributions of fold changes in protein half-life between RPL41 KO and WT cells. Data are shown for all detected proteins (*n* = 4,220) and for MS-down proteins (*n* = 556). The *P*-value was calculated using a two-tailed Mann–Whitney *U* test.

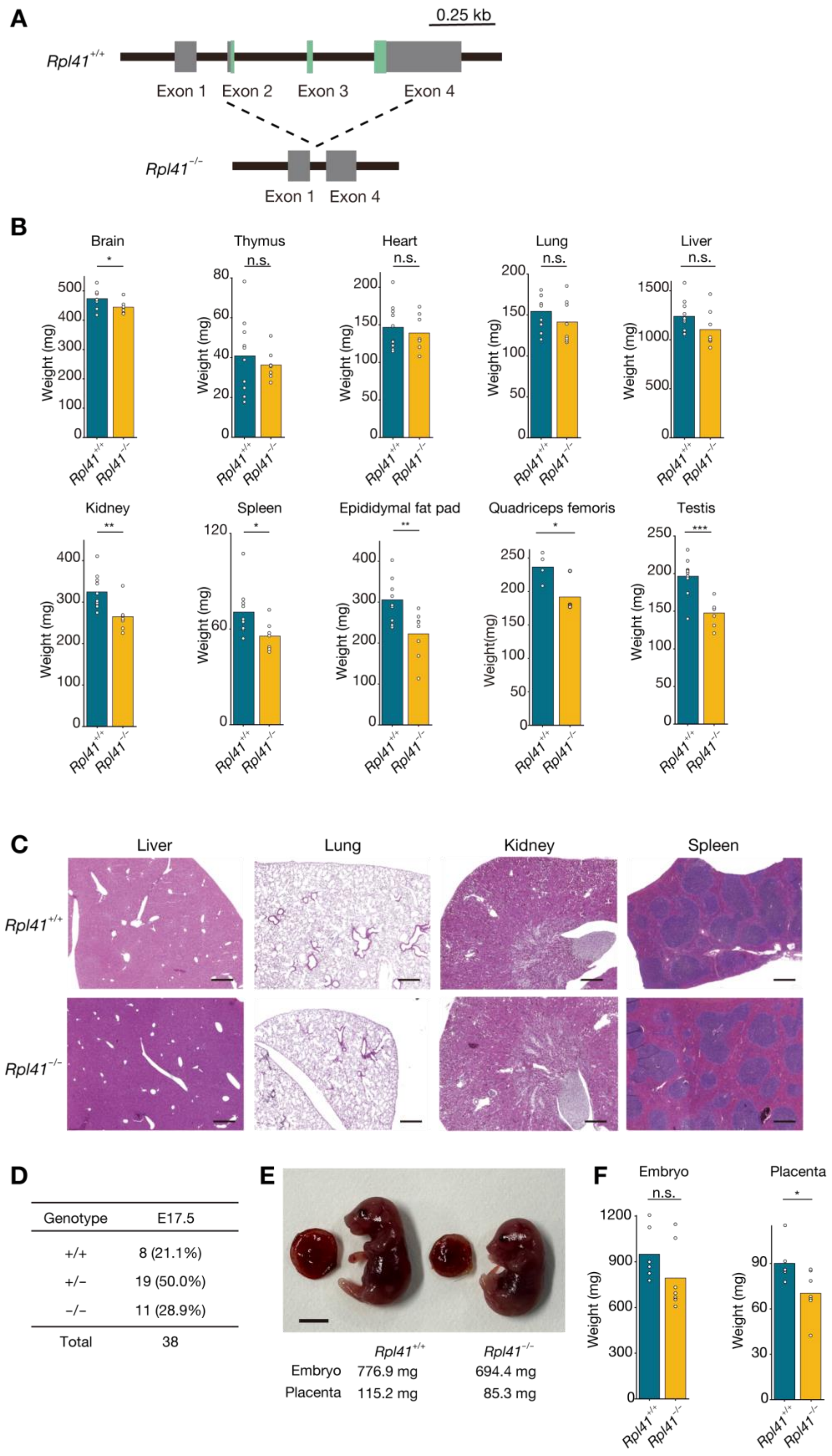

Hirata *et al.* Supplementary Figure 7

Supplementary Figure S7 Phenotypic characterization of *Rpl41*$^{-/-}$ mice.

(**A**) Schematic representation of the wild-type and mutant alleles of mouse Rpl41. Exons are shown as boxes, and the open reading frame is highlighted in green.

(**B**) Tissue weights of the brain, thymus, lung, heart, liver, kidney, epididymal fat pad, spleen, testis, and quadriceps femoris from 10-week-old male *Rpl41*$^{+/+}$ (n = 10 for all tissues except quadriceps femoris, for which n = 4) and *Rpl41*$^{-/-}$ (n = 8 for all tissues except quadriceps femoris, for which n = 4).

(**C**) Representative H&E staining of liver, lung, kidney, and spleen sections from *Rpl41*$^{+/+}$ and *Rpl41*$^{-/-}$ male mice at 10 weeks of age. Scale bars, 400 µm.

(**D**) Genotypic distribution and percentages of offspring at embryonic day 17.5 (E17.5) from heterozygous (*Rpl41*$^{+/-}$) intercrosses.

(**E**) Representative images of fetuses and placentas from *Rpl41*$^{+/+}$ and *Rpl41*$^{-/-}$ mice at E17.5, with corresponding body and placenta weights indicated. Scale bar, 0.5 cm.

(**F**) Body and placenta weights of *Rpl41*$^{+/+}$ (n = 6) and *Rpl41*$^{-/-}$ (n = 7) embryos at E17.5. *P < 0.05, **P < 0.01, ***P <0.001; n.s., not significant (two-tailed Student's t test).

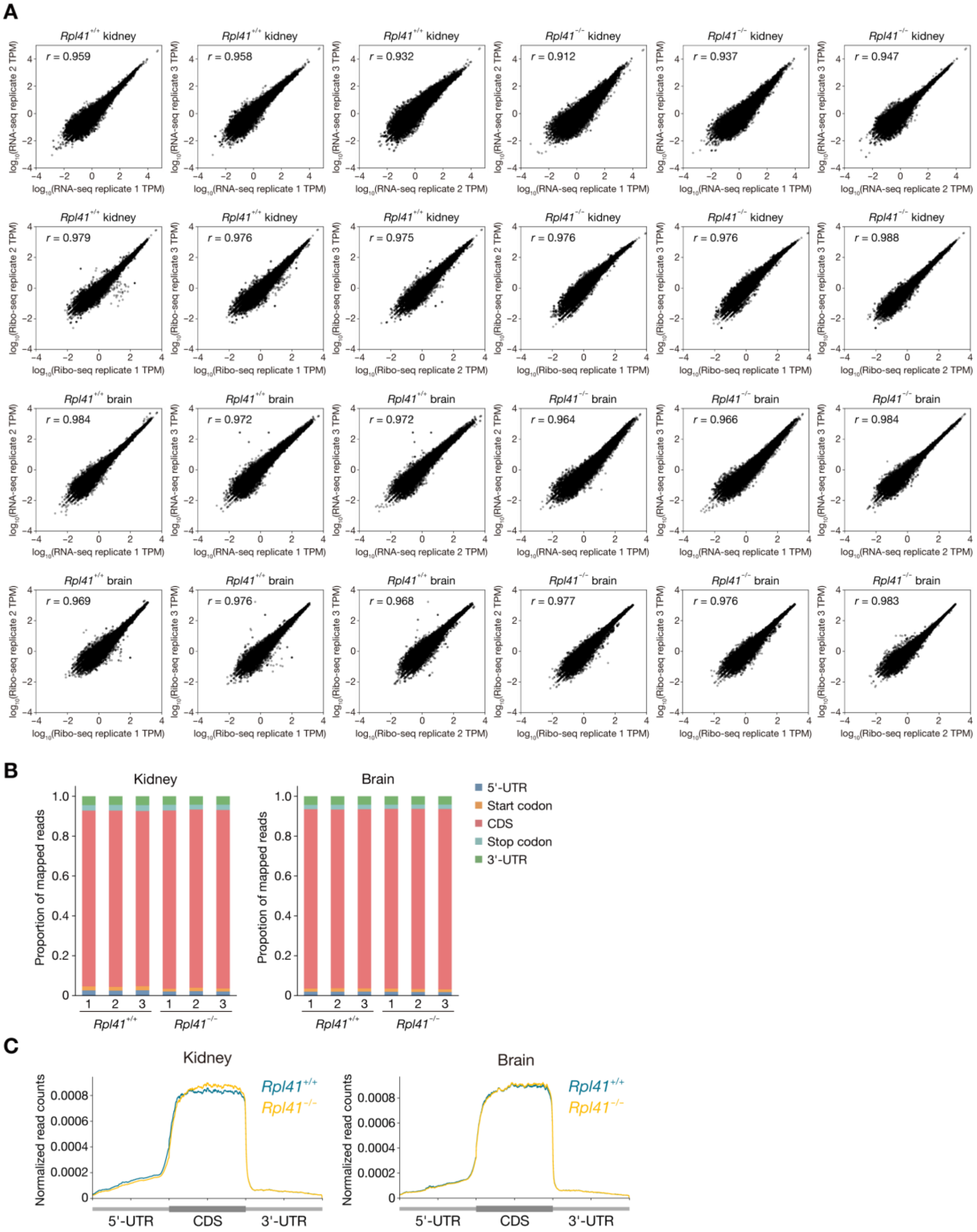
A
Rpl41+/+ kidney
r = 0.959
r = 0.958
r = 0.932
Rpl41−/− kidney
r = 0.912
r = 0.937
r = 0.947
r = 0.979
r = 0.976
r = 0.975
r = 0.976
r = 0.976
r = 0.988
Rpl41+/+ brain
r = 0.984
r = 0.972
r = 0.972
Rpl41−/− brain
r = 0.964
r = 0.966
r = 0.984
r = 0.969
r = 0.976
r = 0.968
r = 0.977
r = 0.976
r = 0.983
log10(RNA-seq replicate 1 TPM)
log10(RNA-seq replicate 2 TPM)
log10(RNA-seq replicate 3 TPM)
log10(Ribo-seq replicate 1 TPM)
log10(Ribo-seq replicate 2 TPM)
log10(Ribo-seq replicate 3 TPM)
B
Kidney
Brain
Proportion of mapped reads
Rpl41+/+
Rpl41−/−
5'-UTR
Start codon
CDS
Stop codon
3'-UTR
C
Kidney
Brain
Normalized read counts
Rpl41+/+
Rpl41−/−
5'-UTR
CDS
3'-UTR

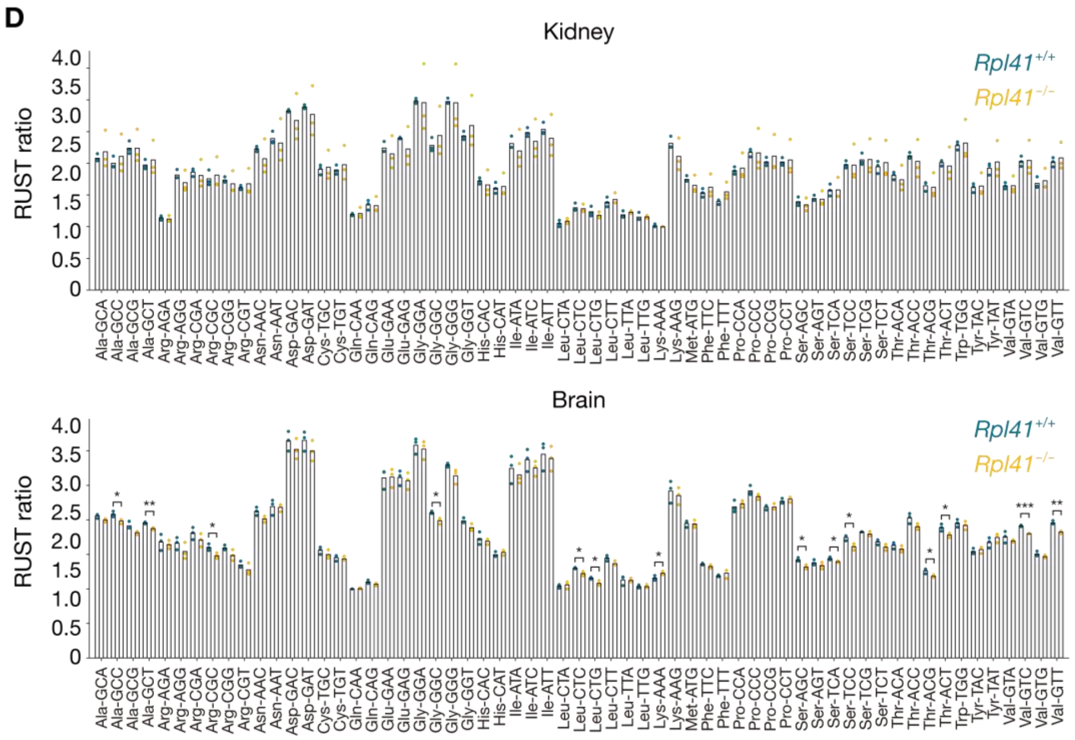

Hirata *et al.* Supplementary Figure 8

Supplementary Figure S8 Ribo-seq quality metrics and codon-level occupancy in *Rpl41*$^{+/+}$ and *Rpl41*$^{-/-}$ mouse tissues.

(**A**) Correlation of TPM values from RNA-seq and Ribo-seq between biological replicates in kidney and brain samples. Pearson's correlation coefficient (r) is indicated.

(**B**) Distribution of ribosome-protected fragments (RPFs) across the 5′-UTR, start codon, coding sequence (CDS), stop codon, and 3′-UTR in kidney and brain of *Rpl41*$^{+/+}$ and *Rpl41*$^{-/-}$ mice (n = 3).

(**C**) Metagene plots of ribosome footprints obtained by Ribo-seq from kidney and brain of *Rpl41*$^{+/+}$ and *Rpl41*$^{-/-}$ mice (n = 3). Data represent the average of three replicates.

(**D**) RUST ratios at the level of individual codons in the kidney and brain of *Rpl41*$^{-/-}$ versus *Rpl41*$^{+/+}$ mice (n = 3). *$P < 0.05$, **$P < 0.01$, ***P <0.001 (two-tailed Student's t test).

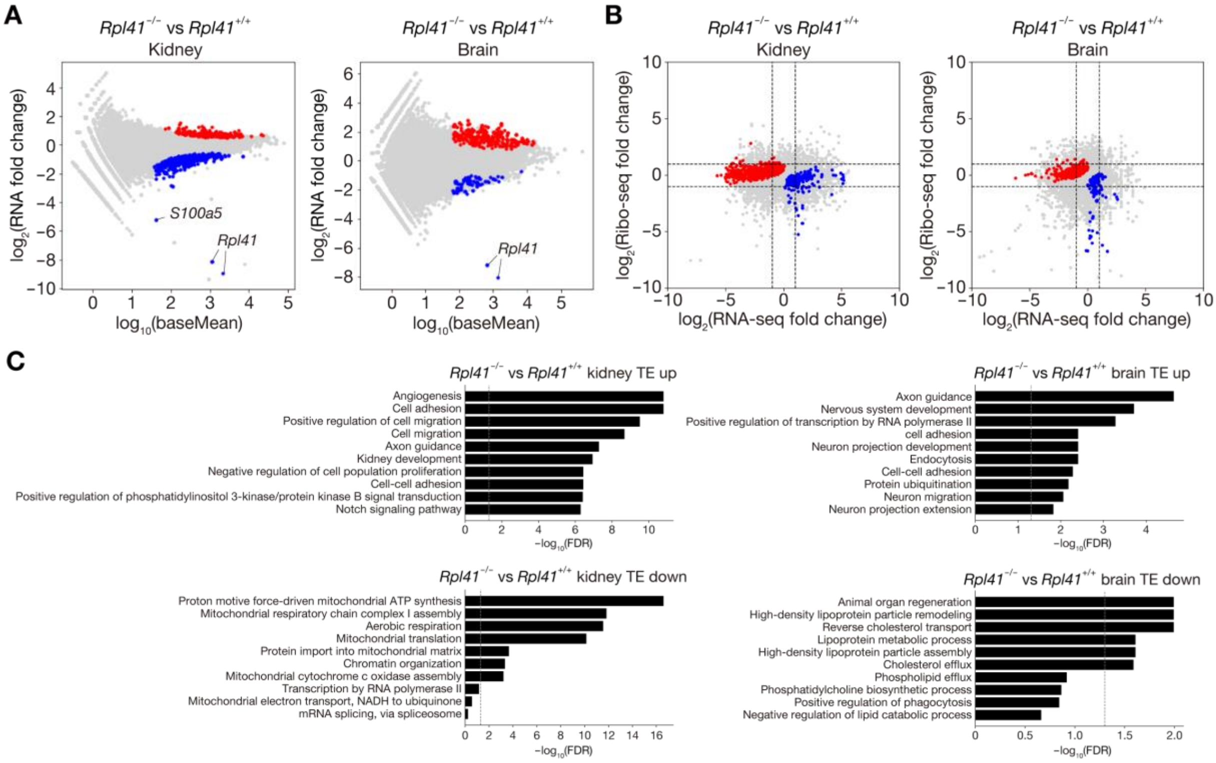

Hirata *et al.* Supplementary Figure 9

Supplementary Figure S9 Transcriptome-wide changes in Rpl41-deficient mouse tissues.

(**A**) MA plots of RNA-seq data in the kidney and brain of *Rpl41*$^{-/-}$ versus *Rpl41*$^{+/+}$ mice (n = 3). Red and blue dots indicate transcripts with significant upregulated or downregulated expression in *Rpl41*$^{-/-}$ compared with the controls.

(**B**) Comparison of fold changes in RNA-seq reads and Ribo-seq footprint reads in *Rpl41*$^{-/-}$ versus control tissues (n = 3). Red and blue dots indicate transcripts with significantly increased or decreased translation efficiency, respectively. Gray dashed lines indicate $\log_2$(fold change) values of ±1.

(**C**) GO analysis of genes showing significantly increased or decreased translation efficiency in *Rpl41*$^{-/-}$ kidney and brain relative to *Rpl41*$^{+/+}$ tissues. *P*-values were calculated as EASE scores (modified Fisher's exact *P* values). FDR, false discovery rate.

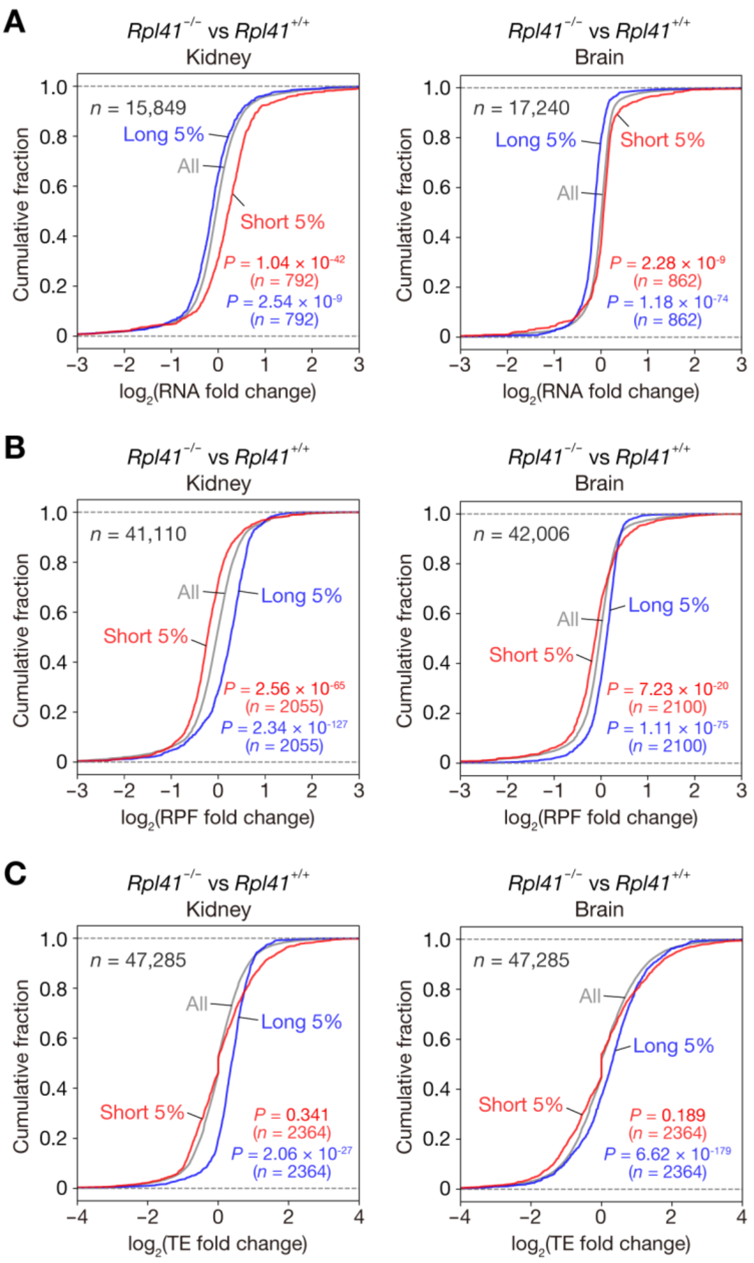

Hirata *et al.* Supplementary Figure 10

Supplementary Figure S10 Length-dependent changes in mRNA abundance, RPF abundance, and translation efficiency in Rpl41-deficient mouse tissues.

(**A**–**C**) Cumulative distributions of fold changes in mRNA abundance (A), RPF abundance (B), and TE (C) in the kidney and brain of *Rpl41*$^{-/-}$ mice relative to *Rpl41*$^{+/+}$ controls (n = 3). Data are shown for all transcripts, the top 5% longest protein-coding transcripts, and the bottom 5% shortest protein-coding transcripts. *P*-values were calculated using two-tailed Mann–Whitney *U* tests.
